# ER-trafficking triggers NRF1 ubiquitination to promote its proteolytic activation

**DOI:** 10.1016/j.isci.2023.107777

**Published:** 2023-08-29

**Authors:** Claire Chavarria, Léa Zaffalon, Sérgio T. Ribeiro, Mélanie Op, Manfredo Quadroni, Maria Sofia Iatrou, Chloé Chapuis, Fabio Martinon

**Affiliations:** 1 Department of Immunobiology, University of Lausanne, 155 Ch. des Boveresses, 1066 Epalinges, Switzerland; 2 Protein Analysis Facility, Center for Integrative Genomics, University of Lausanne, 1015 Lausanne, Switzerland

**Keywords:** Natural sciences, Biological sciences, Biochemistry, Cell biology

## Abstract

The transcription factor NRF1 resides in the endoplasmic reticulum (ER) and is constantly transported to the cytosol for proteasomal degradation. However, when the proteasome is defective, NRF1 escapes degradation and undergoes proteolytic cleavage by the protease DDI2, generating a transcriptionally active form that restores proteostasis, including proteasome function. The mechanisms that regulate NRF1 proteolytic activation and transcriptional potential remain poorly understood. This study demonstrates that the ER is a crucial regulator of NRF1 function by orchestrating its ubiquitination through the E3 ubiquitin ligase HRD1. We show that HRD1-mediated NRF1 ubiquitination is necessary for DDI2-mediated processing in cells. Furthermore, we found that deficiency in both *RAD23A* and *RAD23B* impaired DDI2-mediated NRF1 processing, indicating that these genes are essential components of the DDI2 proteolytic machinery. Our findings highlight the intricate mechanism by which the ER activates NRF1 to coordinate the transcriptional activity of an adaptation response in cells.

## Highlights

• ER trafficking triggers HRD1-mediated ubiquitination of NRF1
• NRF1 ubiquitination is necessary for proteolytic maturation by DDI2
• RAD23 regulates NRF1-mediated proteasome degradation and cleavage via DDI2
• DDI2 contributes to the expression of distinct NRF1 transcriptional programs

## Graphical abstract

**Figure:**
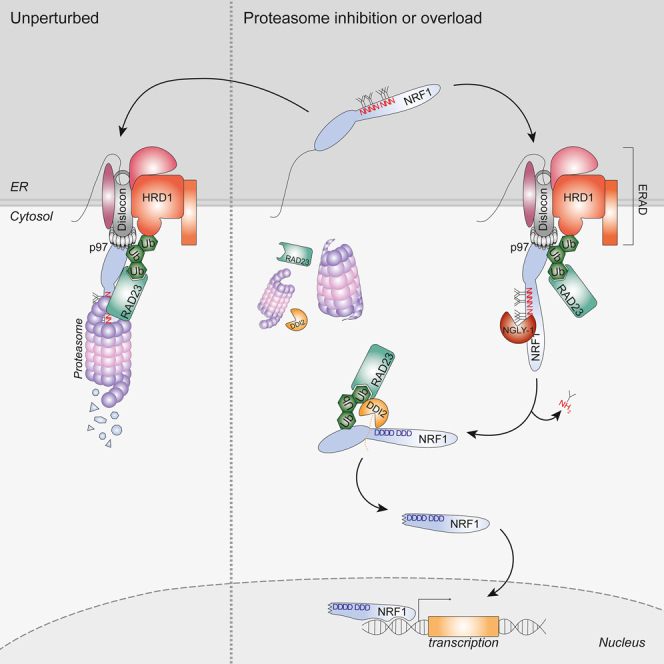

## Introduction

The endoplasmic reticulum (ER) is an organelle conserved in eukaryotes that plays a crucial role in protein synthesis, folding, and trafficking, as well as in other essential cellular functions, such as lipid synthesis, calcium storage, and carbohydrate metabolism.^1^^,^^2^ Moreover, the ER serves as a gateway to the degradation pathway by targeting misfolded proteins for degradation through the ubiquitin-proteasome system (UPS).^3^ This process is known as the ER-associated degradation (ERAD) pathway, whereby misfolded proteins are extracted from the ER lumen and retrotranslocated into the cytosol for degradation.^4^

Considered a central protein folding hub, the ER governs the modifications, structural maturation, and targeted transportation of over one-third of the cellular proteome, including all proteins embedded in membranes or those retained in specific cellular compartments or released extracellularly.^5^ Furthermore, recent evidence suggests that the ER may also be involved in the posttranslational modifications of at least one cytosolic protein, the Cap’n'Collar (CnC) basic leucine zipper (bZIP) transcription factor NFE2L1, also known as NRF1.^6^ In *C. elegans*, the homolog of NRF1, SKN-1A, undergoes N-glycosylation within the ER lumen. In the cytosol, the glycosylated asparagine residues are processed, and the asparagine amino acids edited into aspartic acid residues.^7^ This amino acid change is required to unleash NRF1 transcriptional activity.^7^

The transcription factor NRF1 regulates genes associated with inflammation, oxidative stress response, and other cellular processes in mammals. It does so via the antioxidant response element (ARE) or the Maf recognition element (MARE).^8^ Similar to its homolog NRF2, which initiates an appropriate adaptation response to oxidative stress,^9^ NRF1 can detect various insults and trigger specific transcriptional programs. For example, NRF1 is activated as a response to proteasome impairment to restore proteasomal activity by promoting the transcription of proteasome subunit genes.^10^^,^^11^^,^^12^ Furthermore, inhibition of NRF1 activation has been shown to increases the susceptibility of Multiple Myeloma to proteasome inhibitor-based chemotherapy.^13^^,^^14^^,^^15^^,^^16^ Moreover, NRF1 has been shown to be involved in the physiological response to pollutants such as cadmium.^17^

The stability and proteolytic maturation of the NRF1 protein are crucial steps in controlling its activation as observed upon proteasome system impairment.^18^^,^^19^ Conversely, proteasome inhibition allows NRF1 to escape degradation and orchestrate the transcriptional response that restores proteasome homeostasis.^10^^,^^11^

Another critical step that controls NRF1 activity relies on its proteolytic maturation, which requires the protease DDI2.^20^^,^^21^ However, the mechanisms by which DDI2 contributes to this process are poorly understood. Biochemical experiments have suggested that purified DDI2 could promote the cleavage of high molecular weight ubiquitinated substrates, including NRF1.^15^ The aspartic protease DDI2 can increase proteasome activity^14^^,^^22^ and may bind substrates via ubiquitin chains through its ubiquitin-interacting motif (UIM) and ubiquitin-like domain (UBL).^22^^,^^23^

While NRF1 cleavage has been proposed to require ubiquitination,^24^^,^^25^ cellular experiments have shown that DDI2’s UBL and UIM are dispensable for NRF1 maturation.^16^ This suggests that other domains, including the helical domain (HDD), may be involved in this process. Moreover, evidence in yeast indicates that DDI2 homologs interact with RAD23 proteins through their respective ubiquitin-associated (UBA) domains.^26^ In humans, DDI2 lacks the UBA domain; however, *in vitro*, RAD23 has been shown to enhance DDI2 proteolytic activity,^15^ suggesting that UBL-harboring proteins may cooperate to trigger NRF1 activation in humans.

NRF1 activation also requires p97/VCP, a component of the endoplasmic reticulum (ER) retrotranslocation machinery.^10^^,^^18^ This machinery is involved in the transport of misfolded proteins into the cytosol for degradation, suggesting that trafficking to the ER and subsequent retrotranslocation of NRF1 could precede maturation by cytosolic DDI2.

In this study, we show that ER trafficking function is to tag NRF1 protein for DDI2-mediated proteolytic maturation. Mechanistically, we describe that this process is independent of glycosylation and relies on NRF1 ubiquitination by the E3-ligase HRD1-mediated. The role of NRF1 ubiquitination is also supported by the observation that UBA-harboring RAD23 proteins are required for DDI2-mediated NRF1 activation. Additionally, we demonstrate that ER-dependent glycosylation in human cells plays a DDI2-independent role in promoting the transcriptional activity of NRF1. Moreover, we show that DDI2-mediated cleavage affects the transcriptional program induced by NRF1 but is not required for NRF1’s ability to promote transcription. These findings indicate that posttranslational modifications within the ER are essential and cooperate to coordinate NRF1 transcriptional responses.

## Results

### NRF1 cleavage by DDI2 requires sequences upstream and downstream of the cleavage site

**Figure 1:**
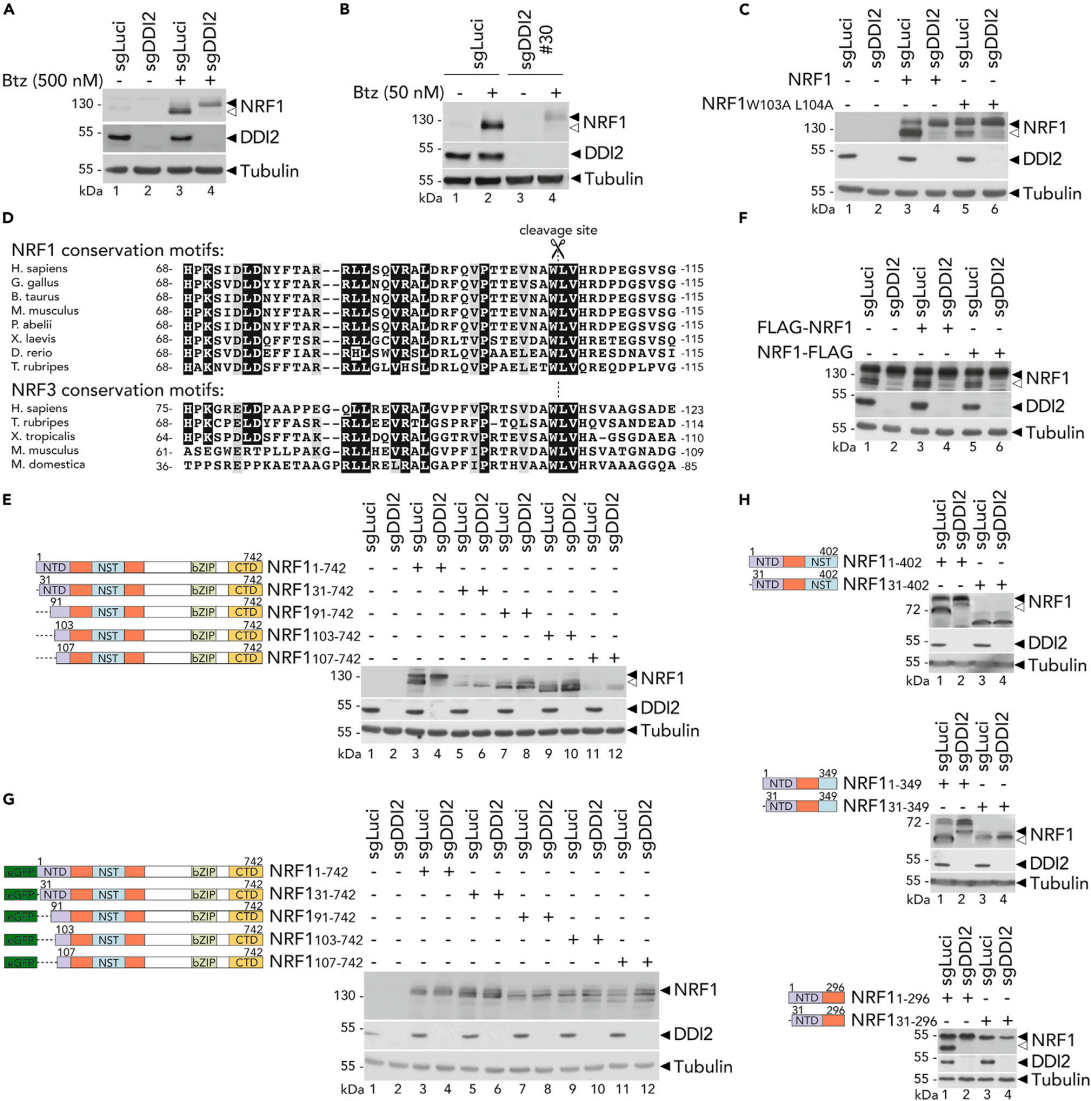
DDI2 mediates NRF1 cleavage at Leucin 104 (A and B) Endogenous NRF1 cleavage in control (sgLuci) and DDI2 knock-out (sgDDI2) HEK293T cells (A) or ARH77 cells (B) treated with Bortezomib (Btz) for 6 h as indicated. Protein expression is measured by Western blot. Tubulin is used as loading control; ◀ indicates the full-length NRF1 protein; ◁ indicates the cleaved NRF1 protein. (C) NRF1 cleavage in control (sgLuci) and DDI2 knock-out (sgDDI2) HEK293T transfected with NRF1 wild-type or cleavage site mutant W103A L104A. Protein expression is measured by Western blot as in A. (D) Sequence alignment of the conserved cleavage site of NRF1 and NRF3 among representative species. (E–H) NRF1 cleavage in control (sgLuci) and DDI2 knock-out (sgDDI2) HEK293T transfected with NRF1 deletion constructs within the N-terminus (E), fused with an FLAG tag at the N-terminus or C-terminus (F) or fused with a eGFP tag at the N-terminus (G), and the C-terminus (H), the C-terminus (H), as illustrated on the left of the panels. NTD, N-terminal domain; NST, Asn/Ser/Thr-rich glycosylated domain; bZIP, Basic Leucine Zipper domain; CTD, C-terminal domain. Protein expression is measured by Western blot as in A. Western blots are representative of three (A, B, E, and F) or two (C, G, and H) independent experiments.

To investigate DDI2-mediated NRF1 cleavage, we generated a DDI2 knock-out population using Crispr-Cas9 technology in human embryonic kidney (HEK) 293T cells. To inhibit NRF1 constitutive degradation by the proteasome,^18^ we treated the cells with Bortezomib (Btz) and monitored DDI2-mediated NRF1 cleavage by immunoblot analysis (Figure 1A). DDI2-mediated processing was also observed in ARH77^16^ and HeLa cells in the presence of proteasome inhibitors (Figures 1B and S1A). Previous studies using Edman degradation-based N-terminal sequencing have shown that NRF1, upon overexpression, was cleaved in N-terminal at Leucine-104.^10^ To confirm that DDI2 targets this site, we mutated the putative cleavage site at residues 103 and 104 (W103A L104A). We confirmed that this mutation partially affected DDI2-dependent NRF1 processing, suggesting that DDI2 could recognize alternative sites within NRF1 (Figure 1C).

To test this hypothesis, we purified FLAG-tagged NRF1 proteins expressed in cells proficient or deficient for DDI2 and investigated the cleavage site by liquid-chromatography mass spectrometry (LC-MS) analysis. We detected only fragments with cleavage between positions 103 and 104 in DDI2-expressing cells (Figure S1B). These data confirm that DDI2 cleaves NRF1 at position 103 and suggest that DDI2 may recognize structural elements in addition to particular amino acid sequences, similar to mechanisms used by the closely related protease from HIV.^27^ Furthermore, phylogenetic comparisons show some conservation in the cleavage site in NRF1, indicating the region’s structural preservation. NRF3, a paralog of NRF1 cleaved by similar mechanisms,^28^ displays similar conservation around the cleavage site (Figure 1D).

To identify the minimal regions required for DDI2-mediated processing, we generated several NRF1 deletion constructs and monitored processing in DDI2 deficient or control cells. We found that deletion within the N-terminal sequence of NRF1 completely abolished processing (Figure 1E). Furthermore, adding an N-terminal FLAG tag did not impact NRF1 processing and showed comparable cleavage as NRF1 fused to an FLAG tag at the C-terminus (Figure 1F). In contrast, linking a 28 kDa eGFP moiety at the N-terminus of NRF1 abrogated its processing (Figure 1G).

To study the involvement of the C-terminal portion of NRF1, we deleted several regions in NRF1 C-terminus. We found that all constructs with sequences shorter than 1–246 could not be cleaved by DDI2 (Figure S1C). Truncated NRF1 protein (amino acids 1–246) showed partial cleavage compared to full-length NRF1. In contrast, construct 1–296 corresponding to the N-Terminal region (NTD) and the intermediated domain preceding the Asn/Ser/Thr-rich glycosylated region (NST), as well as other shorted deletions in the C-terminus showed robust DDI2-mediated cleavage (Figure 1H).

Taken together, these observations indicate that NRF1 processing requires several elements upstream and downstream of its cleavage site, with the N-terminus being essential. Adding a short sequence did not affect the function of the N-terminus; however, a more prominent independent fold could impact its function.

### ER localization is required for NRF1 proteolytic maturation

Given that the N-terminal region of NRF1 was proposed to be involved in anchoring the protein within the ER,^29^^,^^30^^,^^31^ we hypothesized that ER trafficking could be a prerequisite to license NRF1 for DDI2-mediated cleavage in the cytosol.

**Figure 2:**
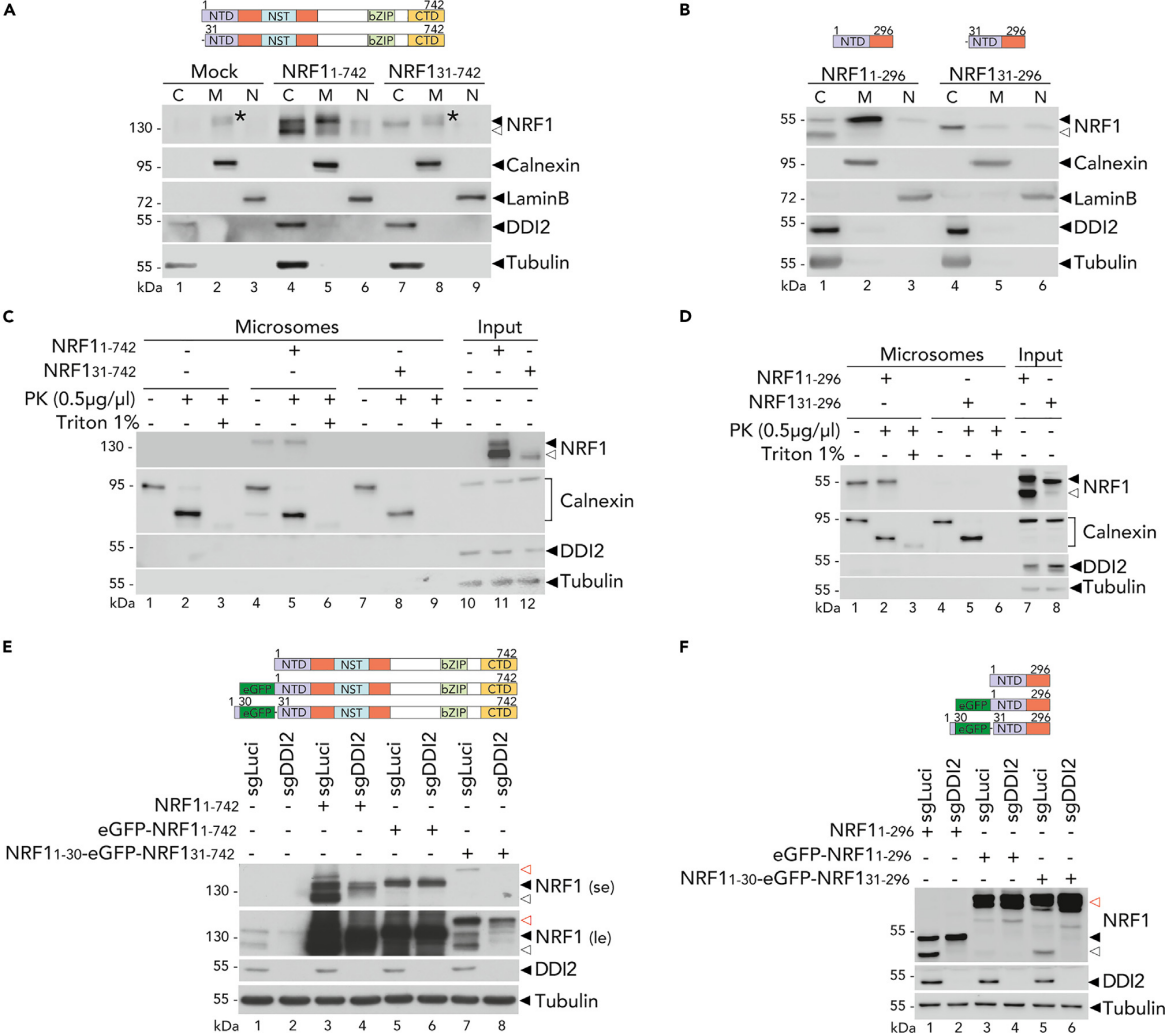
ER-localization of NRF1 is essential for its DDI2-mediated processing (A and B) NRF1 full-length (1–742) or lacking a functional 30NTD (31–742) (A) or NRF1 (1–296) and (31–296) (B) were expressed in HEK293T. Lysates were fractionated by sequential centrifugation into the membrane fraction (M: ER, Golgi, mitochondria), the nucleus (N) and the cytosol (C), respectively. NRF1 was monitored by Western blot. Calnexin, Lamin B and Tubulin are loading and fractionation controls. ∗ indicates endogenous NRF1 protein. (C and D) NRF1 full-length (1–742) or lacking a functional 30NTD (31–742) (C) or NRF1 (1–296) and (31–296) (D) were expressed in HEK293T. Microsomes were isolated as described under “method details, microsome purification assay.” Microsomes were incubated with or without Proteinase K (PK) or Triton X-100 as indicated. NRF1 was monitored by Western blot. Calnexin and Tubulin are loading and microsomes controls. (E and F) NRF1 full-length (1–742), or fused to an N-terminal eGFP tag, or expressing the 30 first amino acids in front of the eGFP tag and lacking a functional 30NTD (E) or similar shorter functional constructs (F) were expressed in control (sgLuci) and DDI2 knock-out (sgDDI2) HEK293T. NRF1 cleavage was monitored by Western blot. red ◁ indicates the unprocessed NRF1 protein fused to an N-terminal eGFP tag; se: short image exposure; le: long image exposure. Tubulin is a loading control. ◀ indicates the unprocessed NRF1 protein and ◁ indicates the cleaved NRF1 protein. Western blots are representative of three (A, B, and E) or two (C, D, and F) independent experiments.

To examine the importance of NRF1 cellular localization, we tested whether the NRF1 N-terminal domain (NTD) is dispensable for DDI2-mediated activation. Structural studies suggested that the region encoding the first 30 amino acids of NRF1 (30NTD) could dictate its entry into the ER.^30^ HEK293T cells expressing wild-type NRF1 (1–742) or lacking its first 30 amino acids (31–742) were fractionated, and proteins were isolated from membrane compartments of the Golgi apparatus, the mitochondria, and the ER (as indicated by the "M" fraction on the immunoblots) (Figure 2A). Transfected full-length NRF1 lacking the 30NTD was poorly detected and overlapped with a signal possibly from endogenous NRF1 in the M fraction. Therefore, to confirm that this 30NTD deleted version is not present in the M fraction, we studied the shorter versions of NRF1 (1–296) and (31–296) and demonstrated that the NRF1 construct lacking the 30NTD was absent from the M fraction, highlighting the importance of this region in mediating NRF1 membrane localization (Figure 2B).

To further examine the role of the NRF1 first 30 amino acids in facilitating ER-trafficking, microsomes were isolated from HEK293T cells expressing wild-type NRF1 (1–742) or lacking the 30NTD (31–742) (Figure 2C) or similar shorter constructs (Figure 2D). Purified microsomes were subjected to proteinase K (PK) treatment to determine the conformation of ER-associated proteins. We monitored Calnexin, an ER-specific type I transmembrane protein as a control. The digestion of microsomes revealed a 70 kDa proteinase-resistant fragment, consistent with the fact that the majority of the Calnexin was intraluminal. Unprocessed NRF1 remained intact after PK treatment in comparison to the Calnexin control, indicating that NRF1 protein is entirely located in the ER lumen before retrotranslocation (Figures 2C and 2D). Differences in protein expression between NRF1 wild-type and NRF1 construct lacking the 30NTD did not allow for a direct comparison. However, we were unable to detect the constructs lacking the 30NTD in the microsome fraction, which is consistent with the possibility that these constructs are mostly cytosolic (Figures 2C and 2D).

In order to examine if ER-localization is a prerequisite for proteolytic activation, the NRF1 region coding for 30NTD was fused to the N-terminus of the eGFP moiety, which abrogated NRF1 cleavage, as shown in Figure 1G. Constructs were then monitored for NRF1 processing in DDI2-deficient and control cells. Fusion of the NRF1 30NTD sequence in front of the eGFP partially restored DDI2-mediated NRF1 cleavage. Both the eGFP NRF1 (31–742) (Figure 2E) and eGFP NRF1 (31–296) (Figure 2F) showed some DDI2-dependent cleavage, suggesting that ER-localization can promote NRF1 processing. The incomplete restoration of cleavage could be attributed to the potential influence of eGFP on trafficking processes, such as cytosolic ERAD-mediated retrotranslocation.

The fractionation assays (Figures 2A and 2B) are consistent with DDI2 being a cytosolic protein. Therefore, in line with previous observations,^10^ we confirmed that NRF1 retrotranslocation into the cytosol is required for DDI2-mediated processing. To assess this question, we treated the cells with NMS-873, an inhibitor of p97/VCP protein. The p97/VCP protein is an essential AAA+ ATPase that contributes to the ERAD pathway.^32^ Upon treatment with the p97 inhibitor, NRF1 cleavage in HEK293T was partially abrogated (Figure S2A). Moreover, endogenous NRF1 cleavage was blocked entirely in ARH77 (Figure S2B). These results indicate that the ER trafficking of NRF1 licenses NRF1 for cleavage by DDI2 in the cytosol. Considering the role of the ER in the post-translational modification of proteins,^5^^,^^33^ we sought to interrogate NRF1 posttranslational modifications and their importance in licensing NRF1 for subsequent processing by DDI2.

### DDI2-mediated NRF1 cleavage requires ubiquitination but occurs independently of its glycosylation state

One of the most frequent ER-initiated modifications is N-glycosylation, which involves attaching a pre-existing sugar chain to an asparagine residue on a newly formed protein. In the glycodomain of NRF1 (NST), there are seven potential asparagine sites that could undergo N-glycosylation.^31^^,^^34^ We investigated the role of N-glycosylations by treating cells with Tunicamycin (TM), an N-glycosylation inhibitor, and monitoring DDI2-mediated NRF1 cleavage via immunoblot analysis. In the presence of TM, DDI2 retained its ability to cleave non-glycosylated NRF1 in ARH77 cells (Figure S3A) and full-length NRF1 protein in HEK293T cells (Figure S3B).

We found that the glycodomain was not required for DDI2 mediated processing of NRF1, as NRF1 constructs with full (1–402), partially deleted (1–349), or completely deleted (1–296) glycodomain were still processed in a DDI2-dependent manner (Figure S3C). Furthermore, it was shown in *C. elegans* that deglycosylation in the cytosol catalyzes a deamidation reaction that releases the glycan moiety and concomitantly converts N-glycosylated asparagine residues to aspartate.^7^ To assess whether this mechanism in mammals contributes to DDI2-mediated NRF1 processing, we engineered NRF1 constructs where the seven glycosylated asparagines are either replaced by aspartic acids (hereafter “7ND”) or alanines to mimic a deglycosylated inactive NRF1 protein (hereafter “7NA”). When expressed in HEK293T cells, both 7ND and 7NA NRF1 mutants are processed in a DDI2-dependent manner (Figure S3D). Altogether these observations indicate that DDI2 mediates NRF1 cleavage independently of the status of its N-glycosylation sites.

To identify other posttranslational modifications (PTMs) of NRF1 that could regulate its processing, we purified FLAG-tagged NRF1 proteins expressed in cells proficient or deficient for DDI2 and analyzed PTMs by LC-MS (liquid chromatography-mass spectrometry) analysis (Figure S4). We detected the presence of one O-glycosylated site at T98, very close to the cleavage site, and two ubiquitinated sites (Figures S3E and S4). First, we investigated the involvement of the O-glycosylation at T98. To test this hypothesis, we monitored DDI2-mediated NRF1 cleavage in DDI2 deficient or control cells treated with OSMI-1, an inhibitor of O-GlcNAc transferase. In the presence of the OSMI-1 inhibitor, NRF1 cleavage was not affected (Figure S3F). Mutation of the O-glycosylated site at residue 98 (T98A) did not affect DDI2-dependent NRF1 processing (Figure S3G), further suggesting that NRF1 is cleaved independently of O-glycosylation.

**Figure 3:**
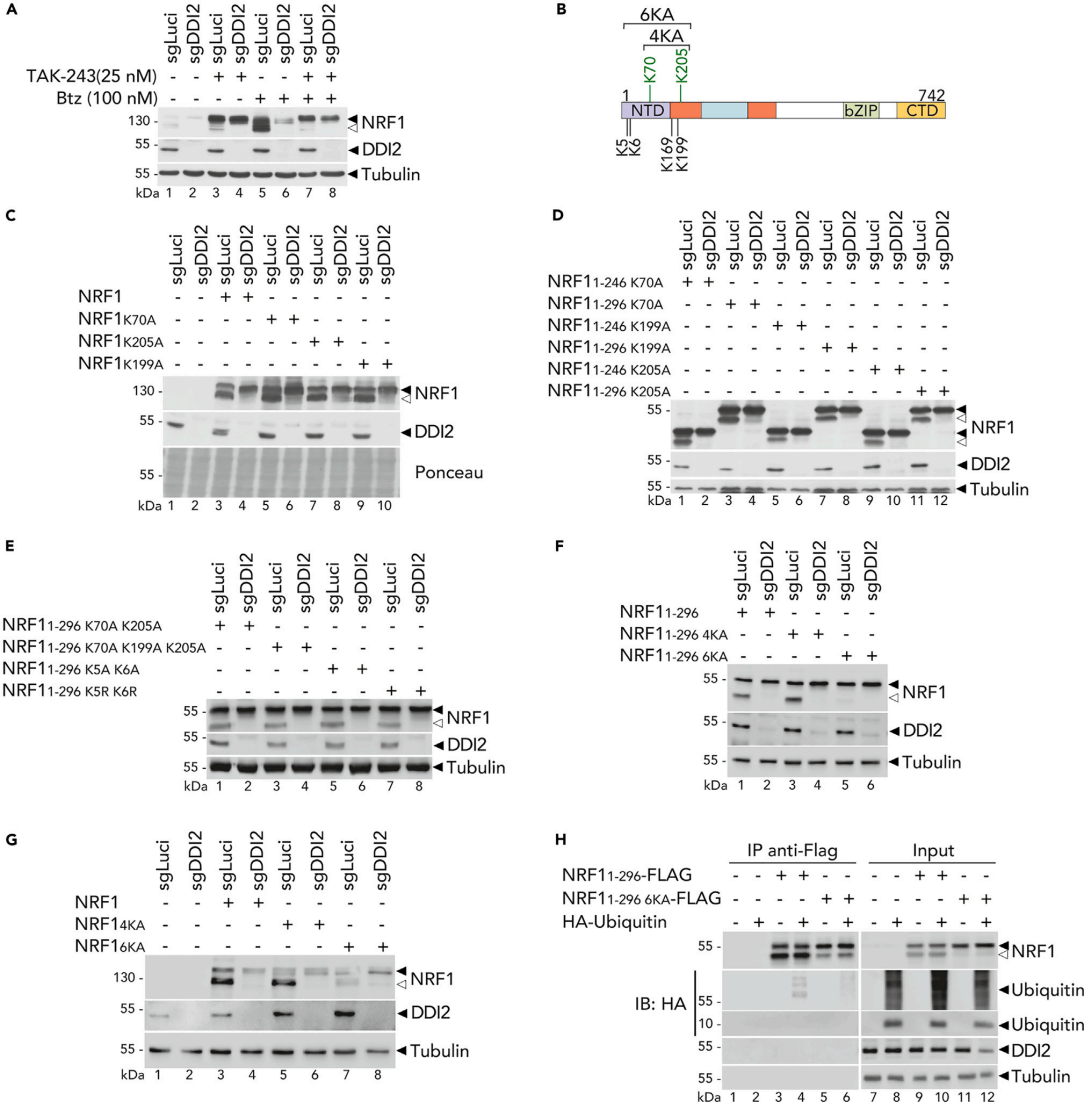
Ubiquitination is essential to mediate NRF1 cleavage (A) Endogenous NRF1 cleavage in control (sgLuci) and DDI2 knock-out (sgDDI2) HeLa cells treated with TAK-243 or/and Bortezomib (Btz) for 6 h as indicated. Protein expression is measured by Western blot. Tubulin is used as loading control; ◀ indicates the full-length NRF1 protein; ◁ indicates the cleaved NRF1 protein. (B) Schematic representation of NRF1 ubiquitination sites (in green); — indicates four lysin residues in NTD of NRF1. (C–G) NRF1 cleavage in control (sgLuci) and DDI2 knock-out (sgDDI2) HEK293T transfected with full-length NRF1 with single ubiquitination site mutants (C), or NRF1 (1–296) or (1–246) (D), or with double ubiquitination site mutants (E), or multiple ubiquitination site mutants (F, G). Protein expression is measured by Western blot. Ponceau or Tubulin is used as a loading control; ◀ indicates the unprocessed NRF1 protein; ◁ indicates the cleaved NRF1 protein. (H) NRF1 immunoprecipitation in HEK293T co-transfected with an HA tagged ubiquitin and Flag-tagged NRF1 (1–296) or (1–296 6KA). Protein expression is monitored by Western blot as in A. Western blots are representative of three (C, D, E, and H) or two (A, F, and G) independent experiments.

Next, we explored the involvement of ubiquitination in NRF1 recognition by DDI2. This hypothesis is supported by previous reports indicating that an inhibitor of ubiquitination affected NRF1 activation and that DDI2 may preferentially recognize large ubiquitylated proteins for degradation by the proteasome.^15^^,^^22^^,^^24^ To investigate the role of NRF1 ubiquitination in DDI2-mediated processing of NRF1, we treated HeLa cells with bortezomib and TAK-243, a UBA1 inhibitor, to block most constitutive ubiquitination. As expected, UBA1 inhibition prevented NRF1 cleavage (Figure 3A). We also generated various mutations within NRF1 lysines residues identified by LC-MS (Figure S4) and four other lysines located within the N-terminus (Figure 3B). Interestingly, when one single lysine mutation is introduced, NRF1 processing was not affected (Figures 3C and 3D). Furthermore, combinations of mutations in the lysine residues also led to no change in NRF1 activation (Figure 3E). In contrast, mutating all six lysine residues within NRF1 N-terminus abolished its DDI2-mediated cleavage (Figures 3F and 3G). These findings suggest that DDI2-mediated NRF1 cleavage does not require a specific lysine residue, but rather any lysine residue that can be ubiquitinated. To confirm the presence of NRF1 ubiquitination, we performed immunoprecipitation of FLAG-tagged NRF1 (1–296) or a version with the six lysine residues mutated (1–296 6KA), in the presence of an HA-tagged ubiquitin moiety. We then monitored ubiquitin conjugation in the pull-downs. As seen in Figure 3H, NRF1 was found to be conjugated with the HA-tagged ubiquitin, whereas NRF1 lacking all six N-terminal lysine residues showed decreased ubiquitination which can result from non-canonical ubiquitination or ubiquitination of the lysines present in the FLAG tag sequence. These results suggest that the lysines within NRF1 N-terminus may play a critical role in directing its ubiquitination and subsequent cleavage by DDI2.

### ER-trafficking of NRF1 licenses its cleavage by promoting its ubiquitination

**Figure 4:**
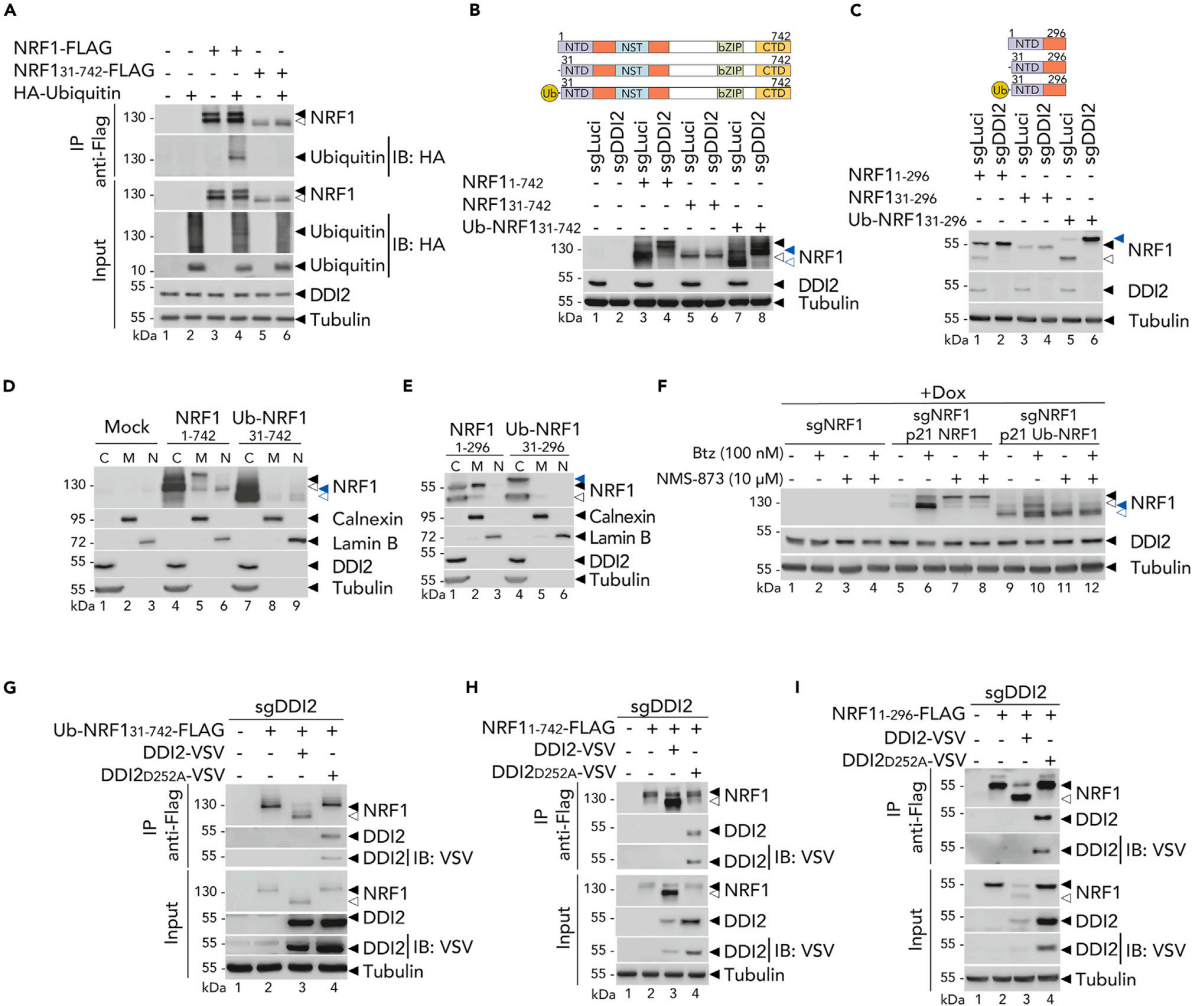
Without trafficking through the ER, ubiquitin-tagged NRF1 is cleaved via conjugation with DDI2 (A) Co-immunoprecipitation in HEK293T co-transfected with an HA tagged ubiquitin and Flag-tagged NRF1 or (31–742). Protein expression is monitored by Western blot. Tubulin is used as a loading control; ◀ indicates the full-length NRF1 protein; ◁ indicates the cleaved NRF1 protein. (B and C) NRF1 cleavage in control (sgLuci) and DDI2 knock-out (sgDDI2) HEK293T transfected with NRF1 (1–742), or (31–742), or fused with a ubiquitin moiety at the N-terminus (B), or similar shorter functional constructs (C) as illustrated on the top of the panels. Protein expression is measured by Western blot. Tubulin is used as a loading control; ◀ indicates the unprocessed NRF1 protein; ◁ indicates the cleaved NRF1 protein; blue ◀ indicates the unprocessed NRF1 protein fused to ubiquitin moiety in N-terminus; blue ◁ indicates the cleaved NRF1 protein fused to ubiquitin moiety in N-terminus. (D and E) NRF1 full-length (1–742) or (31–742) fused to ubiquitin moiety in N-terminus (D) or similar shorter functional constructs (E) were expressed in HEK293T. Lysates were fractionated by sequential centrifugation into the membrane fraction (M: ER, Golgi, mitochondria), the nucleus (N) and the cytosol (C), respectively. Calnexin, Lamin B and Tubulin are loading and fractionation controls. NRF1 was monitored by Western blot as in B. (F) NRF1 cleavage in NRF1 knock-out (sgNRF1) or reconstituted with full-length NRF1 (sgNRF1 p21 NRF1) or NRF1 lacking its functional 30NTD but fused to ubiquitin moiety in N-terminus (sgNRF1 p21 Ub-NRF1) upon doxycycline, in ARH77 cells treated with Btz and/or NMS-873 for 6 h as indicated. Protein expression is monitored by Western blot as in B. (G) Flag-tagged NRF1 (31–742) fused to ubiquitin moiety in N-terminus co-immunoprecipitation with DDI2 wild-type or protease domain mutant (D252A) in HEK293T cells. Protein expression is monitored by Western blot as in A. (H and I) Flag-tagged full-length NRF1 (1–742) or lacking functional 30NTD (31–742) (H), or similar shorter functional constructs (I), co-immunoprecipitation with DDI2 wild-type or protease domain mutant (D252A) in HEK293T cells. Protein expression is monitored by Western blot as in A. Western blots are representative of three (A, B, C, D, E, and F) or two (G, H, and I) independent experiments.

To understand how the trafficking within the ER affects NRF1 ubiquitination, we studied NRF1 constructs that do not traffic to the ER. We previously observed that NRF1 lacking the 30NTD does not localize to the ER (Figures 2A and 2B). Interestingly, we did not observe robust ubiquitination of the NRF1 lacking the 30NTD (Figure 4A), suggesting that ER trafficking is involved in the ubiquitination of NRF1. We used this cytosolic form of NRF1 that is not subject to ubiquitination to investigate whether we could restore the process of proteolytic maturation by fusing a ubiquitin moiety to the N-terminus of the protein. Our results revealed that adding a single ubiquitin fold to NRF1 was sufficient to restore DDI2-mediated cleavage (Figures 4B and 4C). Cell fractionation studies indicated that these constructs were only present in the cytosolic fraction and not in the membrane fraction (Figures 4D and 4E). To confirm that the cytosolic, ubiquitinated form of NRF1 is cleaved independently of ERAD, we monitored NRF1 cleavage in ARH77 cells deficient in NRF1, reconstituted with an inducible form of full-length NRF1 or the cytosolic version (lacking the 30NTD) fused to ubiquitin at its N-terminus. We induced NRF1 expression in the presence of NMS-873. Consistently, this ERAD inhibitor blocked the proteolytic maturation of full-length NRF1 (Figures 4F and S2A). However, NMS-873 did not affect the processing of the Ub-NRF1 construct at basal or upon treatment with bortezomib (Figure 4F). Similarly, we observed that fusion of ubiquitin to NRF1 constructs harboring mutation in their lysines, restored its cleavage (Figure S4B). These findings provide evidence that DDI2 is capable of processing cytosolic proteins directly and that NRF1 localization within the ER main function is to license NRF1 for cleavage by promoting its ubiquitination.

To investigate the role of NRF1 ubiquitination in its recruitment to DDI2, a protease known to interact with ubiquitinated proteins,^15^^,^^22^ we expressed NRF1, lacking its functional 30NTD, and fused with a ubiquitin moiety at the N-terminus, along with either DDI2 or an enzymatic inactive form of DDI2 (D252A), in HEK293T cells deficient in DDI2. We then analyzed NRF1 pull-downs. We observed that only the enzymatically inactive DDI2 was pulled down together with NRF1 (Figures 4G–4I), suggesting that DDI2 binds to full length NRF1 and dissociates right after proteolytic processing. Collectively, these findings indicate that the ubiquitination at the N-terminus of NRF1 is ER-dependent and that this process is required for the recognition and the subsequent processing by DDI2.

### The E3 ligase HRD1 ubiquitinates NRF1 upon retrotranslocation from the ER

The ER plays a critical role in regulating protein quality control and ER-associated degradation (ERAD) pathways through the action of several E3 ligases. One such E3 ligase is the HMG-CoA reductase degradation 1 (HRD1), which is a transmembrane protein involved in the degradation of misfolded ER proteins via the ERAD pathway. Previous studies have suggested that HRD1 may also contribute to the degradation of NRF1 and other misfolded proteins.^15^^,^^19^^,^^35^ To investigate the potential involvement of HRD1 in NRF1 processing, we generated HRD1 knock-out cell lines and assessed the impact of HRD1 deficiency on NRF1 activation.

**Figure 5:**
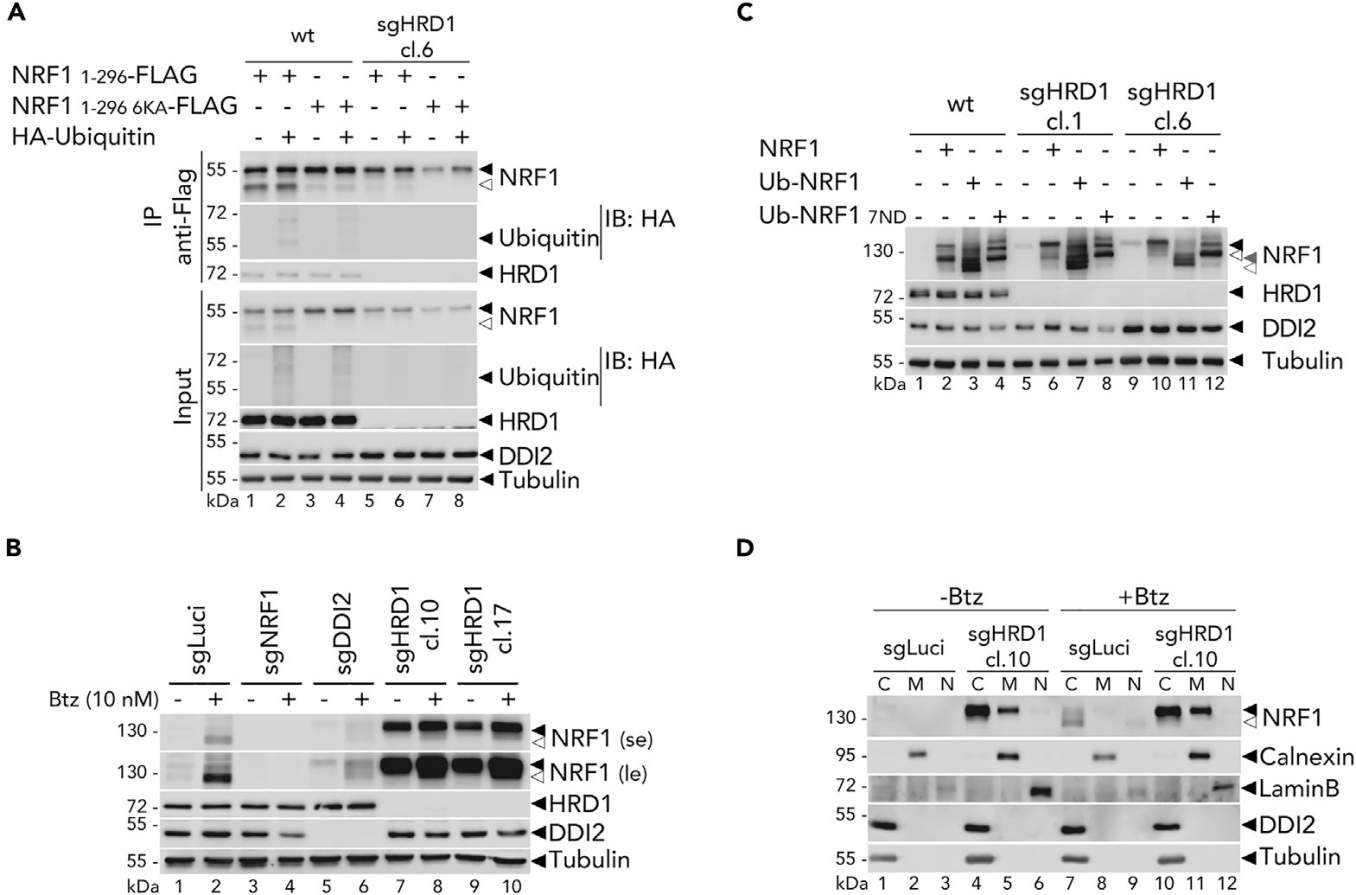
HRD1 mediates NRF1 ubiquitination following its retrotranslocation from the ER (A) Flag-tagged NRF1 (1–296) or (1–296 6KA) immunoprecipitation in wild-type and HRD1 knock-out (sgHRD1 cl.6) HEK293T co-transfected with an HA tagged ubiquitin. Protein expression is monitored by Western blot. Tubulin is used as a loading control; ◀ indicates the full-length NRF1 protein; ◁ indicates the cleaved NRF1 protein. (B) Endogenous NRF1 cleavage in control (sgLuci), NRF1 knock-out (sgNRF1), DDI2 knock-out (sgDDI2) and two clones for HRD1 knock-out (sgHRD1 cl.10 and cl.17) ARH77 cells treated with Btz for 24 h as indicated; se: short image exposure; le: long image exposure. Protein expression is measured by Western blot as in A. (C) NRF1 cleavage in control (sgLuci) and HRD1 knock-out (sgHRD1 cl.1 and cl.6) HEK293T transfected with NRF1 wild-type, or lacking its functional 30NTD and fused to ubiquitin in N-terminus (Ub-NRF1) and with N-glycosylation sites mutations (Ub-NRF1 7ND). Protein expression is measured by Western blot as in A; gray ◀ indicates the unprocessed NRF1 protein fused to ubiquitin moiety in N-terminus; gray ◁ indicates the cleaved NRF1 protein fused to ubiquitin moiety in N-terminus. (D) Endogenous NRF1 localization in control (sgLuci) and HRD1 knock-out (sgHRD1 cl.10) ARH77 treated with Btz for 6 h. Lysates were fractionated by sequential centrifugation into the membrane fraction (M: ER, Golgi, mitochondria), the nucleus (N) and the cytosol (C), respectively. Calnexin, Lamin B and Tubulin are loading and fractionation controls. NRF1 was monitored by Western blot as in A. Western blots are representative of two (D) or one independent experiment.

First, we immunoprecipitated a functional NRF1 construct co-expressed with HA-tag ubiquitin in wild-type and HRD1-deficient HEK293T cells. We observed that the ubiquitination of NRF1 was abrogated in HRD1 knock-out cells (Figure 5A). Furthermore, in ARH77 cells, HRD1 deficiency led to the accumulation of NRF1 and impaired its proteolytic cleavage (Figure 5B). This accumulation of NRF1 was observed in the absence of bortezomib, highlighting the dual role of HRD1 in mediating NRF1 degradation and its requirement for activation.

To demonstrate that HRD1 did not impact DDI2 activity *per se*, we analyzed the proteolytic activation of the cytosolic versions of Ub-NRF1 and Ub-NRF1 7ND. HRD1 deficiency did not impact cytosolic Ub-NRF1 cleavage, indicating that its function is required upstream of DDI2 activation (Figure 5C). Importantly, HRD1 deficiency did not affect NRF1 retrotranslocation in the cytosol, as demonstrated by fractionation studies showing that NRF1 accumulated in the cytosolic fraction of HRD1-deficient ARH77, independently of proteasome inhibition. In contrast, in HRD1-proficient cells, NRF1 is constantly ubiquitinated and degraded which renders its detection only possible upon proteasome inhibition, a condition that promotes its cleavage in the cytosol (Figure 5D).

These experiments indicate that HRD1 is crucial for the ubiquitination of NRF1 at the ER, a key step that licenses NRF1 for the subsequent DDI2-mediated cleavage in the cytosol.

### RAD23 is required for DDI2-mediated NRF1 cleavage

The yeast homolog of DDI2 has a ubiquitin-associated domain (UBA), which mediates interaction with ubiquitinated proteins. However, in humans, DDI2 lacks this fold and reconstitution experiments have shown that neither the ubiquitin-like domain (UBL) nor its C-terminus are required for DDI2 activity.^16^ Interestingly, RAD23, a UBA-containing protein, has been shown to enhance DDI2 proteolytic activity *in vitro*.^15^ In addition, studies in yeast have shown a genetic and physical interaction between yeast DDI1 and RAD23.^36^ Therefore, we hypothesize that RAD23 may contribute to recruit ubiquitinated proteins to DDI2.

**Figure 6:**
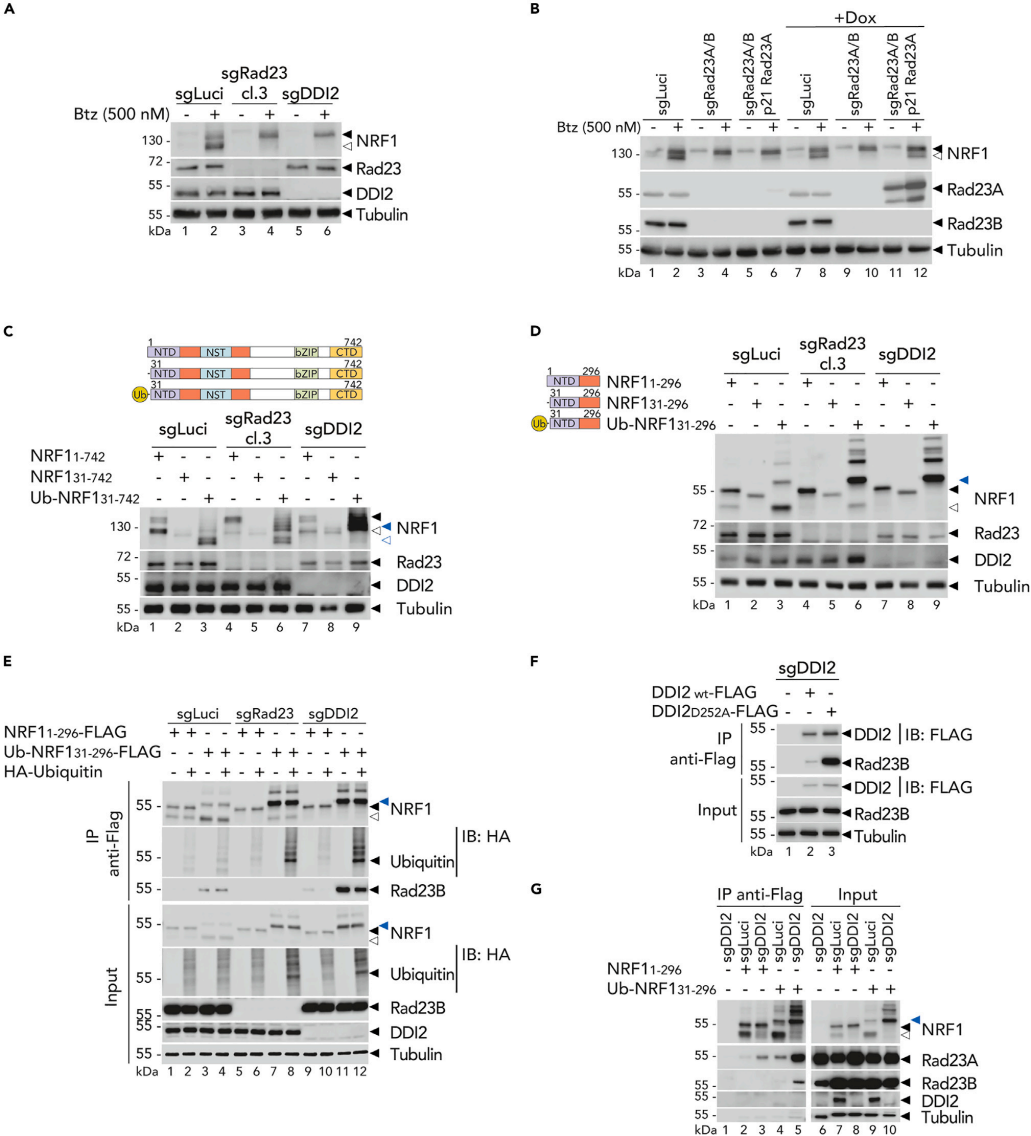
RAD23 conjugates to DDI2 to trigger NRF1 cleavage (A) Endogenous NRF1 cleavage in control (sgLuci), double RAD23 knock-out (sgRad23 cl.3) and DDI2 knock-out (sgDDI2) HEK293T cells treated with Btz for 6 h as indicated. Protein expression is measured by Western blot. Tubulin is used as loading control; ◀ indicates the full-length NRF1 protein; ◁ indicates the cleaved NRF1 protein. (B) NRF1 cleavage in control (sgLuci) or double RAD23 knock-out (sgRad23 A/B) reconstituted with Rad23a (p21 Rad23A), in HEK293T cells treated with Btz for 6 h as indicated. Protein expression is monitored by Western blot as in A. (C and D) NRF1 cleavage in control (sgLuci), double RAD23 knock-out (sgRad23 cl.3) and DDI2 knock-out (sgDDI2) HEK293T transfected with full-length NRF1, NRF1 lacking its functional 30NTD and with ubiquitin moiety fused in N-terminus (C), or with similar shorter functional constructs (D) as illustrated on the left of the panels. Protein expression is measured by Western blot as in A. blue ◀ indicates the unprocessed NRF1 protein fused to ubiquitin moiety in N-terminus; blue ◁ indicates the cleaved NRF1 protein fused to ubiquitin moiety in N-terminus. (E) Flag-tagged NRF1 immunoprecipitation in wild-type (sgLuci), double RAD23 knock-out (sgRad23) and DDI2 knock-out (sgDDI2) HEK293T co-transfected with an HA tagged ubiquitin and short functional NRF1 or (31–296). Protein expression is monitored by Western blot as in A. (F) DDI2 immunoprecipitation in DDI2-deficient HEK293T cells transfected with flag-tagged wild-type DDI2 or DDI2 proteolytically inactive (D252A). Protein expression is measured by Western blot as in A. (G) Flag-tagged NRF1 immunoprecipitation in wild-type (sgLuci) and DDI2 knock-out (sgDDI2) HEK293T transfected short functional NRF1 or Ub-NRF1. Protein expression is monitored by Western blot as in A. Western blots are representative of at least two independent experiments.

To investigate whether human RAD23 proteins are required for NRF1 maturation in cells, we generated RAD23 knock-out HEK293T by targeting the two RAD23 paralogues (Rad23a and Rad23b). We found that double deficiency of Rad23a and Rad23b, similar to DDI2 deficiency, decreased NRF1 cleavage (Figure 6A) whereas single deficiency of either Rad23a or Rad23b did not impaired NRF1 cleavage (Figure S5). Additionally, reconstitution of Rad23a expression in the double RAD23 knock-out HEK293T cells rescued partially NRF1 cleavage upon Bortezomib (Figure 6B). Moreover, RAD23 proteins were required for maximal cleavage of the cytosolic NRF1 construct lacking the 30NTD but fused to the ubiquitin fold (Figures 6C and 6D). In addition, we demonstrated that RAD23 deficiency did not impact NRF1 ubiquitination (Figure 6E). These results indicate that RAD23 functions downstream of NRF1 trafficking to the ER or ubiquitination.

To investigate the possible interaction between RAD23 and DDI2, we expressed DDI2 or a catalytically inactive DDI2 construct in HEK293T cells. We found that Rad23B interacted with both active and inactive DDI2, but the interaction was more robust with inactive DDI2, suggesting that the complex is more stable when DDI2 is inactive (Figure 6F). Additionally, we found that Rad23A and to a lesser extend Rad23B interacted with NRF1, and that this interaction was stabilized in the absence of DDI2 (Figure 6G). Altogether, these findings indicate that RAD23 is a co-factor protein possibly involved in shuttling ubiquitinated NRF1 to DDI2 through its interaction with DDI2.

### N-D protein sequence editing and DDI2 mediated cleavage in the cytosol influence NRF1 transcriptional activity

**Figure 7:**
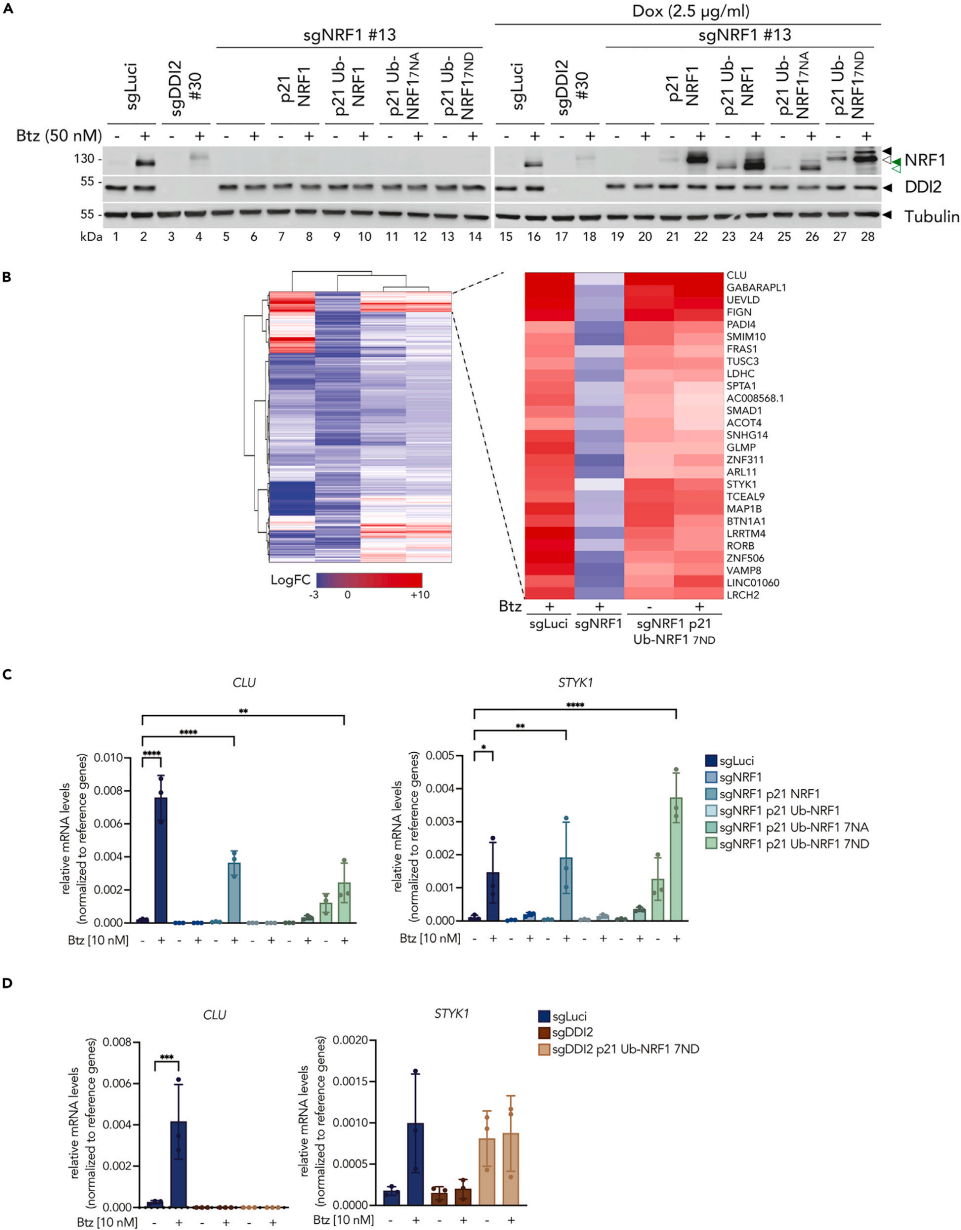
N-D editing and subsequent cytosolic DDI2 cleavage of NRF1 are required to control proteasome gene expression (A) NRF1 cleavage in control (sgLuci), DDI2 knock-out (sgDDI2), NRF1 knock-out (sgNRF1) - or reconstituted with full-length NRF1 (p21 NRF1), or NRF1 lacking its functional 30NTD but fused to ubiquitin moiety in N-terminus (p21 Ub-NRF1), and with N-glycosylation sites mutated into alanine (p21 Ub-NRF1 7NA) or into aspartic acid (p21 Ub-NRF1 7ND) - upon doxycycline, in ARH77 cells treated with Btz for 6 h as indicated. Protein expression is monitored by Western blot. Tubulin is used as a loading control; ◀ indicates the unprocessed NRF1 protein; ◁ indicates the cleaved NRF1 protein; green ◀ indicates the non-glycosylated unprocessed NRF1 protein; green ◁ indicates the non-glycosylated cleaved NRF1 protein. (B) Heatmap showing the NRF1 up-regulated genes in control (sgLuci) and NRF1 knock-out (sgNRF1) reconstituted or not with Ub-NRF1 7ND, ARH77 cells treated with Btz for 24 h. Purified RNA was analyzed for gene expression by RNA-seq. The genes are listed based on hierarchical clustering generated on NG-CHM Builder and with a p-value <0.01. The LogFC is based on the mean from the triplicates from each condition; red color indicates high expression and blue low expression. The right panel shows the more strongly 27 genes upregulated by NRF1. (C) As in A, but treated with Btz for 24 h as indicated. Induction of *STYK1* and *CLU* genes was measured by real-time PCR relative to *GAPDH* and *RPL19* (mean and SEM of technical triplicates of one representative experiment are shown). (D) NRF1 activation in control (sgLuci), DDI2 knock-out (sgDDI2 cl.30), or reconstituted with NRF1 lacking its functional 30NTD but fused to ubiquitin moiety in N-terminus and with N-glycosylation sites mutations (p21 Ub-NRF1 7ND) - upon doxycycline, in ARH77 cells treated with Btz for 24 h as indicated. Induction of *STYK1* and *CLU* genes was measured by real-time PCR relative to *GAPDH* and *RPL19*. Western blots and RT-PCR are representative of three (C, D) and one (A) independent experiments. RT-PCR data are represented as means ± SEM and tested for statistical significance using Sidak’s multiple comparisons test (∗p ≤ 0.05, ∗∗p ≤ 0.01, ∗∗∗p ≤ 0.001, ∗∗∗∗p ≤ 0.0001, are considered significant).

ER signaling contributes to NRF1 activity via two possible mechanisms. First, it promotes the glycosylation and deglycosylation of NRF1, which results in the editing of glycosylated asparagine into aspartic acids. Second, allows for ubiquitination which is essential for NRF1 activation by DDI2. To study how these events contribute to NRF1 transcriptional program, we examined gene expression in NRF1-deficient ARH77 cell line reconstituted with different NRF1 constructs. We used wild-type NRF1, NRF1 lacking its functional N-terminal domain (30NTD) fused to a ubiquitin moiety in the N-terminus (hereafter referred to as "Ub-NRF1"), NRF1 with its seven glycosylation sites replaced by alanines (hereafter referred to as "Ub-NRF1 7NA") or replaced by aspartic acids (hereafter referred to as "Ub-NRF1 7ND"). We induced the expression of the above-mentioned constructs with doxycycline and treated the cells with bortezomib to inhibit the proteasome and thereby trigger NRF1 activation. Upon doxycycline or bortezomib treatment, we confirmed that all NRF1 constructs are cleaved in ARH77 cells (Figure 7A) and confirmed that Ub-NRF1 constructs did not traffic to the ER (Figure S6). The migration of the various products was affected by the glycosylation status and differences in charge observed with the N-D amino acid changes.

To investigate the transcriptional responses, we performed RNA-seq to compare the transcriptional profile of ARH77 control cells (sgLuci), NRF1-deficient (sgNRF1) cells, or (Ub-NRF1 7ND) cells in the presence/absence of bortezomib. Moreover, using the hierarchical clustering method, we were able to detect an overlapping set of genes triggered by bortezomib in an NRF1-dependent manner that are similarly induced in cells expressing the Ub-NRF1 7ND protein (Figure 7B). These data indicate that expression of Ub-NRF1 7ND without proteasome impairment is sufficient to recapitulate the transcriptional programs triggered by NRF1 in the presence of bortezomib.

To verify the activity of the top identified NRF1 targets, we performed real-time qPCR analysis. *STYK1*, and *CLU* gene expression were enriched upon Ub-NRF1 7ND expression, as well as wild-type NRF1 expression (Figure 7C). In contrast, these genes were not induced upon expression of Ub-NRF1 and Ub-NRF1 7NA, indicating that, as previously discovered in *C. elegans*, N-D editing of glycosylated sites is required for optimal transcriptional induction.

The fact that Ub-NRF1 7ND functions independently of treatment with bortezomib offers a unique opportunity to dissect DDI2 cleavage to its activity. To answer this question, we generated DDI2-deficient ARH77 cells expressing Ub-NRF1 7ND. We treated these cells with doxycycline to induce NRF1 expression and performed real-time qPCR analysis in these different ARH77 cell models under bortezomib treatment. We found that while some NRF1-dependent genes such as *CLU* were induced in a DDI2-dependent manner, other genes such as *STYK1* could be induced in a DDI2-independent manner (Figure 7D), indicating that DDI2 engagement may differentially affect NRF1’s transcriptional output.

## Discussion

The behavior of NRF1 is unique as it traffics to the ER before being retranslocated in the cytosol and eventually functions in the nucleus as a transcription factor. This process involves several layers of regulation that orchestrate NRF1 responses. Our study reveals two main regulatory functions of ER trafficking of NRF1 in mammalian cells: NRF1 ubiquitination and N-glycosylation.

Early studies have shown that NRF1 cellular localization was controlled through residues 1–30 within the N-terminus.^30^ It was hypothesized that this sequence could represent an atypical signal sequence that could be regulated during stress in a fashion that ensures its incorporation into the ER at basal.^30^ In line with these initial observations, our studies demonstrated that this sequence is required for NRF1 import in the ER, a process that initiates a cycle of degradation or activation of NRF1. However, the nature of the 30NTD is unclear as it does not contain a typical signal peptidase cleavage site, and the fusion of an FLAG tag at the N-terminus of NRF1 does not perturb its trafficking within the ER. In contrast, we could show that fusing this sequence in front of a cytosolic version of NRF1, harboring an eGFP at the N-terminus, restored both trafficking and DDI2-mediated cleavage. These findings suggest that this sequence alone is sufficient to direct NRF1 import into the ER. It is also possible that this sequence could contribute directly to the retrotranslocation of NRF1 via the ER-associated degradation (ERAD) pathway. However, we cannot rule out the possibility that additional motifs within the first 296 amino acids of NRF1 could also direct its trafficking to the ERAD machinery, as this portion of NRF1 is the minimal structure that traffics in and out of the ER and is required for cleavage by DDI2. The first 296 residues of NRF1 may also contain sequences required for DDI2 recognition. Several attempts at generating an uncleavable version of NRF1 by mutating the region surrounding the cleavage site failed to abolish its cleavage completely. Moreover, attempts at identifying other potential cleavage sites by mass spectrometry only identified the cleavage site between residues 103 and 104. It is, therefore, possible that DDI2 recognition may rely on additional features within the 296 first amino acids. In line with this possibility, it is worth noting that DDI2 proteolytic domain shares homology with that of HIV-1 aspartyl protease, which is known to recognize structural shapes of its substrates rather than a particular amino acid sequence.^37^

Importantly, we describe that NRF1 trafficking within the ER is a prerequisite for DDI2-mediated processing via ubiquitination by HRD1. This ERAD associated E3-ligase has been implicated in various physiological processes, including the ubiquitination of NRF1 homolog, NRF2, upon ER stress during liver cirrhosis.^38^ Here we found that HRD1 deficiency impaired NRF1 ubiquitination without affecting its retrotranslocation leading to its accumulation in the cytosol. In addition to its function in promoting degradation at basal levels, we show that HRD1-mediated ubiquitination is also required for NRF1 proteolytic maturation by DDI2 when proteasome is impaired. We also showed that a cytosolic version of NRF1 fused to a ubiquitin moiety can bypass this step and undergo DDI2-mediated cleavage in an HRD1-independent manner. Altogether these findings suggests that the accumulation of ubiquitylated NRF1 substrate in the cytosol is critical to elicit NRF1 responses.

Previous studies have shown that DDI2 activity on NRF1 was observed only upon the addition of recombinant RAD23 *in vitro*.^15^ Our study using human knockout cells demonstrates that both RAD23 paralogues are necessary for both NRF1-mediated proteasome degradation and NRF1 proteolytic maturation by DDI2. RAD23 acts as a ubiquitin receptor that binds the proteasome via its N-terminal ubiquitin folds and two UBA domains that can bind ubiquitinated proteins.^14^^,^^39^^,^^40^ It functions as a shuttle or receptor to bring substrates to the proteasome and promote turnover. Interestingly, we observed that RAD23 binding to DDI2 was stabilized in the absence of DDI2 catalytic activity, suggesting that the stalling of uncleaved substrates with the complex stabilized the interaction. Overall, our findings support a model where the same mechanisms involved in NRF1 proteasomal degradation, such as HRD1-dependent ubiquitination and RAD23-mediated proteasome targeting, are also engaged, and required in promoting DDI2-mediated NRF1 cleavage.

The second step that occurs upon trafficking in the ER is glycosylation. This was described in a breakthrough study in *C. elegans* that identified the editing by the cytosolic N-glycanase PNG1 of glycosylated asparagine residues into aspartic acid.^7^ Using several mutated constructs within the glycosylation residues, we showed that neither glycosylation nor editing of these residues impacted NRF1 processing by DDI2. In contrast, we observed that the editing of glycosylated asparagine into aspartic acid increased NRF1 transcriptional activity similar to that described in *C. elegans*. These observations suggest that this process is conserved in mammals and further defines an additional regulatory function of ER trafficking. We took advantage of this knowledge to investigate the transcriptional activity of NRF1 in cells expressing an ubiquitinated and edited NRF1 construct that does not traffic to the ER. We identified genes triggered by this cytosolic NRF1 that overlapped with the signature elicited by wild-type NRF1 in the presence of bortezomib. This finding confirmed that editing and ubiquitination were sufficient to bypass ER trafficking and restore transcriptional activity. Consistently, we observed that several of the NRF1-induced genes, including *clusterin* (*CLU*), were induced in a DDI2-dependent manner and therefore relied on the proteolytic maturation of NRF1. *CLU* was identified as among the most NRF1-dependent genes. In contrast, we observed that some of the genes that were identified as NRF1-dependent did not rely on DDI2. For example, the kinase *STYK1* was induced by treatment with bortezomib or expression of an ubiquitinated and edited NRF1 construct in a DDI2-independent manner, suggesting that DDI2 engagement could contribute to determining NRF1 transcriptional programs. Investigation of these different programs and their physiological relevance and implication within NRF1 responses and adaption programs is an important question that remains to be addressed.

In summary, this study delineates the importance of ER trafficking for NRF1 to exert its functions. We demonstrated that the editing of the N-glycosylated sites contributes to transcriptional activity and that HRD1-mediated NRF1 ubiquitination contributes to degradation as well as recognition by the RAD23-DDI2 pathway leading to the expression of distinct transcriptional program. The DDI2-dependent NRF1 pathway has been mostly proposed to contribute to susceptibility and resistance to treatments with proteasome inhibitors in multiple myeloma.^13^^,^^14^^,^^15^^,^^16^ The identification of these additional steps and novel proteins involved in the pathway such as HRD1 and RAD23 could shed a new light on the mechanisms of adaptation and resistance to proteasome inhibition and provide with new therapeutic targets in these diseases.

### Limitations of the study

This study has some limitations. The majority of the investigations were conducted on cellular models through knock-out and reconstitution experiments. To have a clearer understanding of the physiological significance of the pathways identified, it would be beneficial to confirm the findings in an *in vivo* model. Additionally, developing a robust cell-free assay could help to dissect the biochemical steps involved in the recognition of NRF1 by DDI2 and RAD23 and provide a better understanding of the complex being formed.

## STAR★Methods

### Key resources table

**Table:** 

REAGENT or RESOURCE	SOURCE	IDENTIFIER
**Antibodies**		

See Table S1.		

**Chemicals, peptides, and recombinant proteins**		

Doxycycline hyclate	Sigma-Aldrich	Cat# D9891
MG-132 (Z-Leu-Leu-Leu-al)	Sigma-Aldrich	Cat# C2211
OSMI-1	Sigma-Aldrich	Cat# SML1621
NMS-873	Sigma-Aldrich	Cat# SML1128
Tunicamycin	Enzo Life Sciences	Cat# BML-CC104-0010
Puromycin	Enzo Life Sciences	Cat# BML-GR312-0250
Bortezomib	LC Laboratories	Cat# B-1408
TAK-243	Lucerna-chem	Cat# HY-100487
CP-26	Anawa	Cat# AOB13238-1
Proteinase K	Roche	Cat# 03115887001

**Critical commercial assays**		

Phusion High-Fidelity PCR kit	New England BioLabs	Cat# E0553
Pfu DNA Polymerase	Promega	Cat# M774A
2X Reverse Transcription master mix	Applied Biosystems	Cat# 4368814

**Deposited data**		

Raw sequencing data RNA-seq	NCBI	BioProject ID PRJNA897493
Raw mass spectrometry data	ProteomeXchange Consortium	ID PXD041331 10.6019/PXD041331
Raw data for immunoblots	Mendeley	https://doi.org/10.17632/dcxmrzdkvk.1

**Experimental models: Cell lines**		

HEK293T (Female)	Jürg Tschopp	N/A
ARH77 (Female)	Pascal Schneider	N/A
HeLa (Female)	Pascal Schneider	N/A
See Table S2.		

**Oligonucleotides**		

See Table S3.		

**Recombinant DNA**		

pCR3	Pascal Schneider	N/A
pDONR-221	Thermo Fisher Scientific	Cat# 12536017
pINDUCER-21	Stephen Elledge and Thomas Westbrook	N/A
pLentiCRISPRv2-Puro	Sanjana et al.^42^	RRID:Addgene_52961

**Software and Algorithms**		

CRISPRseek Bioconductor package	Zhu et al.^43^	https://doi.org/10.18129/B9.bioc.CRISPRseek

### Resource availability

#### Lead contact

Any additional information or inquiries regarding code availability or resources should be directed to Fabio Martinon (Fabio.Martinon@unil.ch).

#### Materials availability

Request for generated models and constructs should be directed to Fabio Martinon (Fabio.Martinon@unil.ch).

### Experimental model and subject details

HEK293T (Human Embryonic Kidney 293T) and HeLa cells were cultured in DMEM (Gibco Dulbecco’s Modified Eagle Medium) supplemented with 10% v/v FBS (Fetal Bovine Serum, Gibco) at 37°C with 5% CO_2_ and routinely passaged 2 times a week. ARH77 plasma cell leukemia cells were provided by Prof. Pascal Schneider, University of Lausanne, Switzerland. ARH77 cells were cultured in RPMI-1640 (Gibco Roswell Park Memorial Institute) supplemented with 10% v/v FBS, at 37°C with 5% CO_2_ and routinely passaged 2 times a week. Cell Line Authentication was performed for ARH77 used in the study using highly-polymorphic short tandem repeat loci (STRs) (Microsynth). All cell lines were confirmed to be mycoplasma-free.

### Method details

#### Plasmids and molecular biology

Most DDI2 and NRF1 constructs (N-terminal and C-terminal deletions, C-terminal FLAG tag, N-terminal eGFP tag) were generated by PCR amplification using the Phusion High-Fidelity PCR kit and restriction enzyme cloning into a pCR3 backbone. The NRF1 30NTD (1–30 amino acids) was annealed and cloned into a pCR3-derived NRF1 construct. NRF1 and DDI2 point mutants were generated on pCR3-derived NRF1 and DDI2 expression constructs by Pfu DNA Polymerase or by standard double PCR approach. NRF1 7NA and 7ND were generated by subcloning a synthetic pre-annealed oligo, designed and ordered at Biomatik, into a pCR3-derived NRF1 construct. Ub-NRF1 derived constructs were generated by attachment of one ubiquitin moiety (with substitution of the final C-terminal glycine of ubiquitin with a valine residue) in-frame to the N-terminus of NRF1 with pCR3-derived NRF1 constructs. Single guide RNA (sgRNA) sequences for DDI2, NRF1, Rad23A, Rad23B and HRD1 were annealed, Esp3I-digested (Biolabs), gel-purified (Cytiva kit), and ligated into pLentiCRISPRv2-Puro^42^ and pLentiCRISPRv2-Blast for sgRad23B, using T4 DNA ligase (Thermo Scientific). All mutations were verified by sequencing.

#### Generation cell lines

Gene knock-out cell lines were generated by viral transduction of pLentiCRISPRv2^42^ vector containing the sgRNA sequence and a puromycin selection marker. The sgRNA sequence of the Luciferase gene was used as control (sgLuci). Positive populations were selected with 2–3 μg/mL puromycin and/or blasticidin for 15 days. Clones were tested by Western blot for each protein knock-out level. Gene-targeted single guide RNA sequences were designed using the CRISPRseek package of Bioconductor (version 3.6) on R. DDI2 knock-out and NRF1 knock-out ARH77 cells were infected with pINDUCER-21 lentiviruses containing the different NRF1 constructs inducible upon doxycycline treatment and a GFP selection marker. GFP-positive cells were FACS sorted 5 days following infection.

#### Single guide RNA design

Gene-targeted single guide RNA sequences were designed using the CRISPRseek package of Bioconductor (version 3.6) on R.

#### Transient transfection and/or pharmacological treatment

200′000 HEK293T cells/ml were seeded in a 12-multiwells plate. The next day, adherent HEK293T cells were transfected with 1 μg of DNA per well with plasmid of interest as indicated along with eGFP expression vector used as a control for transfection. 24 h following transfection, eGFP expression in HEK293T cells was assessed by fluorescent microscopy and, cells were lysed for subsequent SDS-PAGE analysis or treated with a pharmacological agent prior to lysis. HEK293T, HeLa and ARH77 cell line were treated with indicated concentration of Btz or NMS-873 for 6 h. Drug concentrations and timing was optimized to detect NRF1 maturation while minimizing toxicity and cell death.

#### Immunoblotting

Cell lysates were either lysed directly in Laemmli buffer 4X (10% glycerol, 2% SDS, 50 mM Tris-HCl pH 6.8, 12.5 mM EDTA, 0.02% Bromophenol Blue) complemented with 100 mM of dithiothreitol (DTT) or prepared with ice-cold RIPA buffer (50 mM NaCl, 50 mM Tris pH 7.4, 1 mM EDTA, 0.1% SDS, 1% NP-40, 1% sodium deoxycholate) supplemented with protease inhibitor cocktail (Roche, Basel, Switzerland), 10 mM Na_3_VO_4_, 50 mM NaF, 10 mM Na_4_P_2_O_7_, and 5 μM MG132. Protein extracts were denatured, and equal amounts were separated by SDS-PAGE and transferred to nitrocellulose blotting membranes (Amersham).

#### Quantitative real-time PCR

Total RNA from cells was extracted with PRImeZOL (Canvax, #AN1100) and cDNA was synthetized using 2X Reverse transcription master mix (Applied Biosystems, Waltham, MA, USA) according to manufacturers’ protocols. For quantitative real-time PCR (RT-PCR), SYBR Green fluorescent reagent and LightCycler 480 Real-Time PCR System (Roche) were used. All RT-PCR were performed in experimental triplicate. Primer sequences are listed in Table EV1.

#### Immunoprecipitation assay

HEK293T cells were lysed in lysis buffer containing 0.2% NP-40, 20 mM Tris-HCl pH 7.4, 150 mM NaCl, supplemented with protease inhibitors (cocktail from Roche) and phosphatase inhibitors (Naf, Na_4_P_2_O_7_ and Na_3_VO_4_). Lysates were precleared for 30 min with Sepharose beads (6B100, Sigma-Aldrich), then anti-FLAG agarose beads (Anti-FLAG M2 A2220 Sigma-Aldrich) were added, followed by incubation for 2 h at 4°C. The immunocomplexes were then washed three times with lysis buffer, resuspended in 4X Laemmli buffer and analyzed by Western Blot.

#### Cell fractionation assay

Confluent HEK293T or ARH77 cells grown in 10 cm^2^ Petri dish or T25 flask were washed once with cold PBS 1X. Cells were permeabilized in lysis buffer A (150 mM NaCl, 50 mM HEPES pH 7.4, digitonin 25 μg/mL, 1M Hexylene glycol) for 20 min at 4°C on a rotating wheel. Following centrifugation, supernatants corresponding to cytosolic fraction were recovered. Pellets were then washed twice and lysed in lysis buffer B (150 mM NaCl, 50 mM HEPES pH 7.4, 1% NP-40, 1M Hexylene glycol) for 30 min on ice. Subsequent centrifugation followed and supernatants corresponding to membrane fraction were withdrawn. Pellets corresponding to nucleic fraction were lysed in lysis buffer C (150 mM NaCl, 50 mM HEPES pH 7.4, 0.1% SDS, 0.5% sodium deoxycholate, 1M Hexylene glycol) supplemented with Benzonase. All buffers were complemented with protease inhibitors (cocktail from Roche). The samples were mixed with 4X Laemmli buffer and analyzed by Western Blot.

#### Microsome purification assay

HEK293T cells, grown in 10 cm^2^ Petri dish up to 90% confluency, were washed twice, resuspended in 10 mM HEPES-KOH pH 7.5 buffer and incubated on ice for 10 min. Swollen cells were then sedimented, resuspended in homogenization buffer (10 mM HEPES-KOH pH 7.5, 10 mM KCl, 1.5 mM MgCl2, 5 mM EGTA, and 250 mM sucrose) and passed through a 27G syringe needle for five to 10 times. Homogenates were subjected to serial centrifugations at 600xg (10 min), 3000xg (10 min) and 100,000xg (60 min). Microsomes collected at the end of the ultracentrifugation step were resuspended in membrane buffer (10 mM HEPES-KOH pH 7.5, 50 mM KOAc, 2 mM Mg(OAc)_2_, 1 mM DTT, and 250 mM sucrose). Microsomes were mock-treated or subjected to Proteinase K (0.5 μg/μL) treatment either in the absence or presence of 1% Triton X-100 for 1 h on ice. Samples were then precipitated with trichloroacetic acid (TCA), resuspended in 4X Laemmli buffer and analyzed by Western blot.

#### High-throughput sequencing

For RNA sequencing, RNA was extracted using RNeasy mini kit (Qiagen) from three independent wells of different ARH77 cell lines treated with 2.5 μg/mL of doxycycline and treated or not with 10 nM Btz. High-throughput sequencing was performed at the Lausanne Genomics Technologies Facility (University of Lausanne) on the Illumina HiSeq 2500 using TruSeq SBS Kit v3 reagents. For the RNA-seq analysis, we used a moderated *t*-test from the R bioconductor package “limma” (R version 3.1.1, limma version 3.20.8). The “adjusted p-value” corresponds to the p-values corrected for multiple testing using the Benjamini–Hochberg method.

#### Analysis of NRF1 cleavage site and post-translational modification sites by mass spectrometry

Large-scale precipitation of proteins from control (sgLuci) and DDI2 knock-out (sgDDI2) HEK293T cell lysates transfected with FLAG-tagged NRF1 was done using anti-FLAG M2 agarose beads (see immunoprecipitation assay section), followed by SDS-PAGE and staining with colloidal Coomassie. Bands corresponding to NRF1 in control and DDI2 knock-out cells were excised, and after in-gel digested with trypsin and chloroacetamide as alkylating reagent. Samples were analyzed by liquid chromatography – mass spectrometry (protein analysis facility at Lausanne University).

### Quantification and statistical analysis

Data from one representative independent experiment is shown. All experiments were performed two or three times, except some adaptation experiments. Statistical significances were determined using Graph Pad Prism version 9. The error bars are the standard deviation of the sample.

## Data Availability

• The raw data for the RNA-seq presented in this publication have been deposited in: https://www.ncbi.nlm.nih.gov/sra/PRJNA897493 and are publicly available as of the date of publication. • The mass spectrometry proteomics data have been deposited to the ProteomeXchange Consortium via the PRIDE^41^ partner repository with the dataset identifier PXD041331 and https://doi.org/10.6019/PXD041331 and are publicly available as of the date of publication. • Raw data for immunoblots were deposited on Mendeley: https://doi.org/10.17632/dcxmrzdkvk.1 and are and are publicly available as of the date of publication. This paper does not report original code. Any additional information required to reanalyze the data reported in this paper is available from the lead contact upon request.

## References

[1] Lynes E.M., Simmen T. (2011). Urban planning of the endoplasmic reticulum (ER): how diverse mechanisms segregate the many functions of the ER. Biochim. Biophys. Acta.

[2] Sitia R., Meldolesi J. (1992). Endoplasmic reticulum: a dynamic patchwork of specialized subregions. Mol. Biol. Cell.

[3] Christianson J.C., Ye Y. (2014). Cleaning up in the endoplasmic reticulum: ubiquitin in charge. Nat. Struct. Mol. Biol..

[4] Olzmann J.A., Kopito R.R., Christianson J.C. (2013). The mammalian endoplasmic reticulum-associated degradation system. Cold Spring Harbor Perspect. Biol..

[5] Stevenson J., Huang E.Y., Olzmann J.A. (2016). Endoplasmic Reticulum-Associated Degradation and Lipid Homeostasis. Annu. Rev. Nutr..

[6] Ruvkun G., Lehrbach N. (2023). Regulation and Functions of the ER-Associated Nrf1 Transcription Factor. Cold Spring Harbor Perspect. Biol..

[7] Lehrbach N.J., Breen P.C., Ruvkun G. (2019). Protein Sequence Editing of SKN-1A/Nrf1 by Peptide:N-Glycanase Controls Proteasome Gene Expression. Cell.

[8] Motohashi H., O'Connor T., Katsuoka F., Engel J.D., Yamamoto M. (2002). Integration and diversity of the regulatory network composed of Maf and CNC families of transcription factors. Gene.

[9] Tebay L.E., Robertson H., Durant S.T., Vitale S.R., Penning T.M., Dinkova-Kostova A.T., Hayes J.D. (2015). Mechanisms of activation of the transcription factor Nrf2 by redox stressors, nutrient cues, and energy status and the pathways through which it attenuates degenerative disease. Free Radic. Biol. Med..

[10] Radhakrishnan S.K., den Besten W., Deshaies R.J. (2014). p97-dependent retrotranslocation and proteolytic processing govern formation of active Nrf1 upon proteasome inhibition. Elife.

[11] Radhakrishnan S.K., Lee C.S., Young P., Beskow A., Chan J.Y., Deshaies R.J. (2010). Transcription factor Nrf1 mediates the proteasome recovery pathway after proteasome inhibition in mammalian cells. Mol. Cell.

[12] Roeten M.S.F., Cloos J., Jansen G. (2018). Positioning of proteasome inhibitors in therapy of solid malignancies. Cancer Chemother. Pharmacol..

[13] Chen T., Ho M., Briere J., Moscvin M., Czarnecki P.G., Anderson K.C., Blackwell T.K., Bianchi G. (2022). Multiple myeloma cells depend on the DDI2/NRF1-mediated proteasome stress response for survival. Blood Adv..

[14] Collins G.A., Goldberg A.L. (2020). Proteins containing ubiquitin-like (Ubl) domains not only bind to 26S proteasomes but also induce their activation. Proc. Natl. Acad. Sci. USA.

[15] Dirac-Svejstrup A.B., Walker J., Faull P., Encheva V., Akimov V., Puglia M., Perkins D., Kümper S., Hunjan S.S., Blagoev B. (2020). DDI2 Is a Ubiquitin-Directed Endoprotease Responsible for Cleavage of Transcription Factor NRF1. Mol. Cell.

[16] Op M., Ribeiro S.T., Chavarria C., De Gassart A., Zaffalon L., Martinon F. (2022). The aspartyl protease DDI2 drives adaptation to proteasome inhibition in multiple myeloma. Cell Death Dis..

[17] Ribeiro S.T., de Gassart A., Bettigole S., Zaffalon L., Chavarria C., Op M., Nugraha K., Martinon F. (2022). The protease DDI2 regulates NRF1 activation in response to cadmium toxicity. iScience.

[18] Steffen J., Seeger M., Koch A., Krüger E. (2010). Proteasomal degradation is transcriptionally controlled by TCF11 via an ERAD-dependent feedback loop. Mol. Cell.

[19] Tsuchiya Y., Morita T., Kim M., Iemura S.i., Natsume T., Yamamoto M., Kobayashi A. (2011). Dual regulation of the transcriptional activity of Nrf1 by beta-TrCP- and Hrd1-dependent degradation mechanisms. Mol. Cell Biol..

[20] Koizumi S., Irie T., Hirayama S., Sakurai Y., Yashiroda H., Naguro I., Ichijo H., Hamazaki J., Murata S. (2016). The aspartyl protease DDI2 activates Nrf1 to compensate for proteasome dysfunction. Elife.

[21] Lehrbach N.J., Ruvkun G. (2016). Proteasome Dysfunction Triggers Activation of SKN-1A/Nrf1 by the Aspartic Protease DDI-1. Elife.

[22] Collins G.A., Sha Z., Kuo C.L., Erbil B., Goldberg A.L. (2022). Mammalian Ddi2 is a shuttling factor containing a retroviral protease domain that influences binding of ubiquitylated proteins and proteasomal degradation. J. Biol. Chem..

[23] Sivá M., Svoboda M., Veverka V., Trempe J.F., Hofmann K., Kožíšek M., Hexnerová R., Sedlák F., Belza J., Brynda J. (2016). Human DNA-Damage-Inducible 2 Protein Is Structurally and Functionally Distinct from Its Yeast Ortholog. Sci. Rep..

[24] Sha Z., Goldberg A.L. (2014). Proteasome-mediated processing of Nrf1 is essential for coordinate induction of all proteasome subunits and p97. Curr. Biol..

[25] Sha Z., Goldberg A.L. (2016). Reply to Vangala et al.: Complete inhibition of the proteasome reduces new proteasome production by causing Nrf1 aggregation. Curr. Biol..

[26] Bertolaet B.L., Clarke D.J., Wolff M., Watson M.H., Henze M., Divita G., Reed S.I. (2001). UBA domains mediate protein-protein interactions between two DNA damage-inducible proteins. J. Mol. Biol..

[27] Pettit S.C., Simsic J., Loeb D.D., Everitt L., Hutchison C.A., Swanstrom R. (1991). Analysis of retroviral protease cleavage sites reveals two types of cleavage sites and the structural requirements of the P1 amino acid. J. Biol. Chem..

[28] Zhang Y., Kobayashi A., Yamamoto M., Hayes J.D. (2009). The Nrf3 transcription factor is a membrane-bound glycoprotein targeted to the endoplasmic reticulum through its N-terminal homology box 1 sequence. J. Biol. Chem..

[29] Wang W., Chan J.Y. (2006). Nrf1 is targeted to the endoplasmic reticulum membrane by an N-terminal transmembrane domain. Inhibition of nuclear translocation and transacting function. J. Biol. Chem..

[30] Zhang Y., Lucocq J.M., Yamamoto M., Hayes J.D. (2007). The NHB1 (N-terminal homology box 1) sequence in transcription factor Nrf1 is required to anchor it to the endoplasmic reticulum and also to enable its asparagine-glycosylation. Biochem. J..

[31] Zhang Y., Ren Y., Li S., Hayes J.D. (2014). Transcription factor Nrf1 is topologically repartitioned across membranes to enable target gene transactivation through its acidic glucose-responsive domains. PLoS One.

[32] Lim P.J., Danner R., Liang J., Doong H., Harman C., Srinivasan D., Rothenberg C., Wang H., Ye Y., Fang S., Monteiro M.J. (2009). Ubiquilin and p97/VCP bind erasin, forming a complex involved in ERAD. J. Cell Biol..

[33] Schwarz D.S., Blower M.D. (2016). The endoplasmic reticulum: structure, function and response to cellular signaling. Cell. Mol. Life Sci..

[34] Tomlin F.M., Gerling-Driessen U.I.M., Liu Y.C., Flynn R.A., Vangala J.R., Lentz C.S., Clauder-Muenster S., Jakob P., Mueller W.F., Ordoñez-Rueda D. (2017). Inhibition of NGLY1 Inactivates the Transcription Factor Nrf1 and Potentiates Proteasome Inhibitor Cytotoxicity. ACS Cent. Sci..

[35] Wu X., Siggel M., Ovchinnikov S., Mi W., Svetlov V., Nudler E., Liao M., Hummer G., Rapoport T.A. (2020). Structural basis of ER-associated protein degradation mediated by the Hrd1 ubiquitin ligase complex. Science.

[36] Bertolaet B.L., Clarke D.J., Wolff M., Watson M.H., Henze M., Divita G., Reed S.I. (2001). UBA domains of DNA damage-inducible proteins interact with ubiquitin. Nat. Struct. Biol..

[37] Prabu-Jeyabalan M., Nalivaika E., Schiffer C.A. (2002). Substrate shape determines specificity of recognition for HIV-1 protease: analysis of crystal structures of six substrate complexes. Structure.

[38] Wu T., Zhao F., Gao B., Tan C., Yagishita N., Nakajima T., Wong P.K., Chapman E., Fang D., Zhang D.D. (2014). Hrd1 suppresses Nrf2-mediated cellular protection during liver cirrhosis. Genes Dev..

[39] Wade S.L., Auble D.T. (2010). The Rad23 ubiquitin receptor, the proteasome and functional specificity in transcriptional control. Transcription.

[40] Kim H.T., Goldberg A.L. (2018). UBL domain of Usp14 and other proteins stimulates proteasome activities and protein degradation in cells. Proc. Natl. Acad. Sci. USA.

[41] Perez-Riverol Y., Bai J., Bandla C., García-Seisdedos D., Hewapathirana S., Kamatchinathan S., Kundu D.J., Prakash A., Frericks-Zipper A., Eisenacher M. (2022). The PRIDE database resources in 2022: a hub for mass spectrometry-based proteomics evidences. Nucleic Acids Res..

[42] Sanjana N.E., Shalem O., Zhang F. (2014). Improved vectors and genome-wide libraries for CRISPR screening. Nat. Methods.

[43] Zhu L.J., Holmes B.R., Aronin N., Brodsky M.H. (2014). CRISPRseek: a bioconductor package to identify target-specific guide RNAs for CRISPR-Cas9 genome-editing systems. PLoS One.

