# Supplementary material for: ER-trafficking triggers NRF1 ubiquitination to promote its proteolytic activation
Source: iScience. 2023 Aug 29;26(10):107777. doi: 10.1016/j.isci.2023.107777 (PMC10502413; doi:10.1016/j.isci.2023.107777)
Supplement: Document S1. Figures S1–S6 and Tables S1–S3 [file mmc1.pdf]

iScience, Volume *26*

Supplemental information

ER-trafficking triggers NRF1 ubiquitination

to promote its proteolytic activation

Claire Chavarria, Léa Zaffalon, Sérgio T. Ribeiro, Mélanie Op, Manfredo Quadroni, Maria Sofia Iatrou, Chloé Chapuis, and Fabio Martinon

Supplementary information

Figure S1. DDI2 mediates NRF1 cleavage at Leucine 104, related to Figure 1.

Figure S2. DDI2 mediates NRF1 cleavage upon ER-retrotranslocation blockade, related to Figure 2.

Figure S3. NRF1 is cleaved independently of its glycosylation state, related to Figure 3.

Figure S4. NRF1 is ubiquitinated at lysin 70 and 205, related to Figure 3 and S3.

Figure S5. NRF1 cleavage requires both RAD23 paralogues, related to Figure 6.

Figure S6. Ub-NRF1 constructs are cleaved in the cytosol, related to Figure 7.

Table S1. List of antibodies, related to STAR Methods.

Table S2. List of cell lines, related to STAR Methods.

Table S3. List of oligonucleotides, related to STAR Methods.

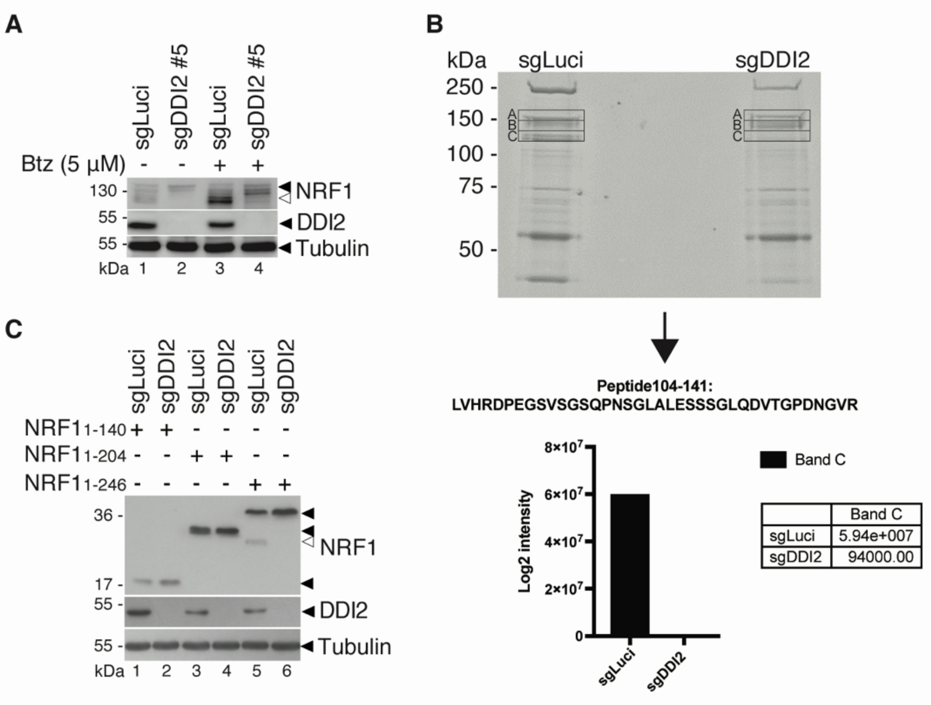

Supplemental figure 1. DDI2 mediates NRF1 cleavage at Leucine 104, related to Figure 1. (A) NRF1 cleavage in control (sgLuci) and DDI2 knock-out (sgDDI2) HeLa cells treated with Bortezomib (Btz) for six hours as indicated. Protein expression is monitored by western blot. Tubulin is used as loading control; ◀ indicates the unprocessed protein; ◁ indicates the cleaved protein. (**B)** NRF1 immunoprecipitation in control (sgLuci) and DDI2 knock-out (sgDDI2) HEK293T cells. Immunoprecipitated samples were loaded on 9% SDS-PAGE gel (top panel). Three bands per condition corresponding to full-length (A and B) and cleaved (C) NRF1 were cut off and digested with trypsin for subsequent LC-MS analysis. The increase of the characteristic peptide 104-141 (bottom panel) in fraction C of control (sgLuci) and not in DDI2 knock-out (sgDDI2) points out a cleavage site between residues 103 and 104. **(C)** NRF1 cleavage in control (sgLuci) and DDI2 knock-out (sgDDI2) HEK293T transfected with NRF1 (1-140) or (1-204) or (1-246). Protein expression is monitored as in A.

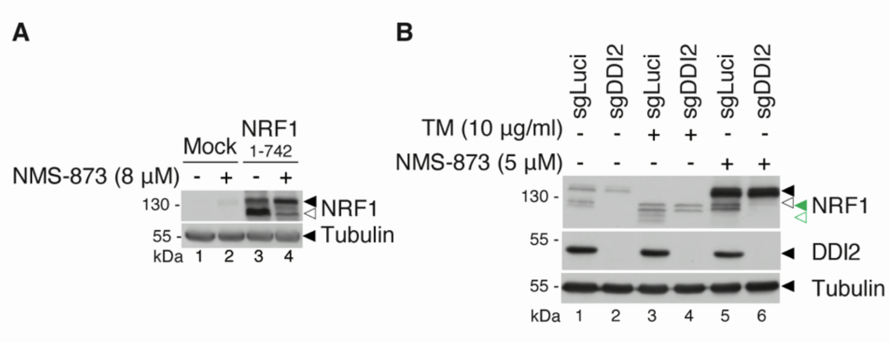

Supplemental figure 2. DDI2 mediates NRF1 cleavage upon ER-retrotranslocation blockade, related to Figure 2. (A) NRF1 cleavage in HEK293T transfected with full-length NRF1 (1-742) and treated with NMS-873 for six hours as indicated. Protein expression is measured by western blot; ◀ indicates the unprocessed protein; ◁ indicates the cleaved protein. **(B)** Endogenous NRF1 cleavage in control (sgLuci) and DDI2 knock-out (sgDDI2) ARH77 cells treated with the N-glycosylation inhibitor, Tunicamycin (TM) or NMS-873, an inhibitor of VCP/97 for six hours as indicated; ◀ indicates the deglycosylated unprocessed protein; ◁ indicates the deglycosylated cleaved protein. Protein expression is measured as in A. Protein expression is monitored as in A. Western blots are representative of at least two independent experiments.

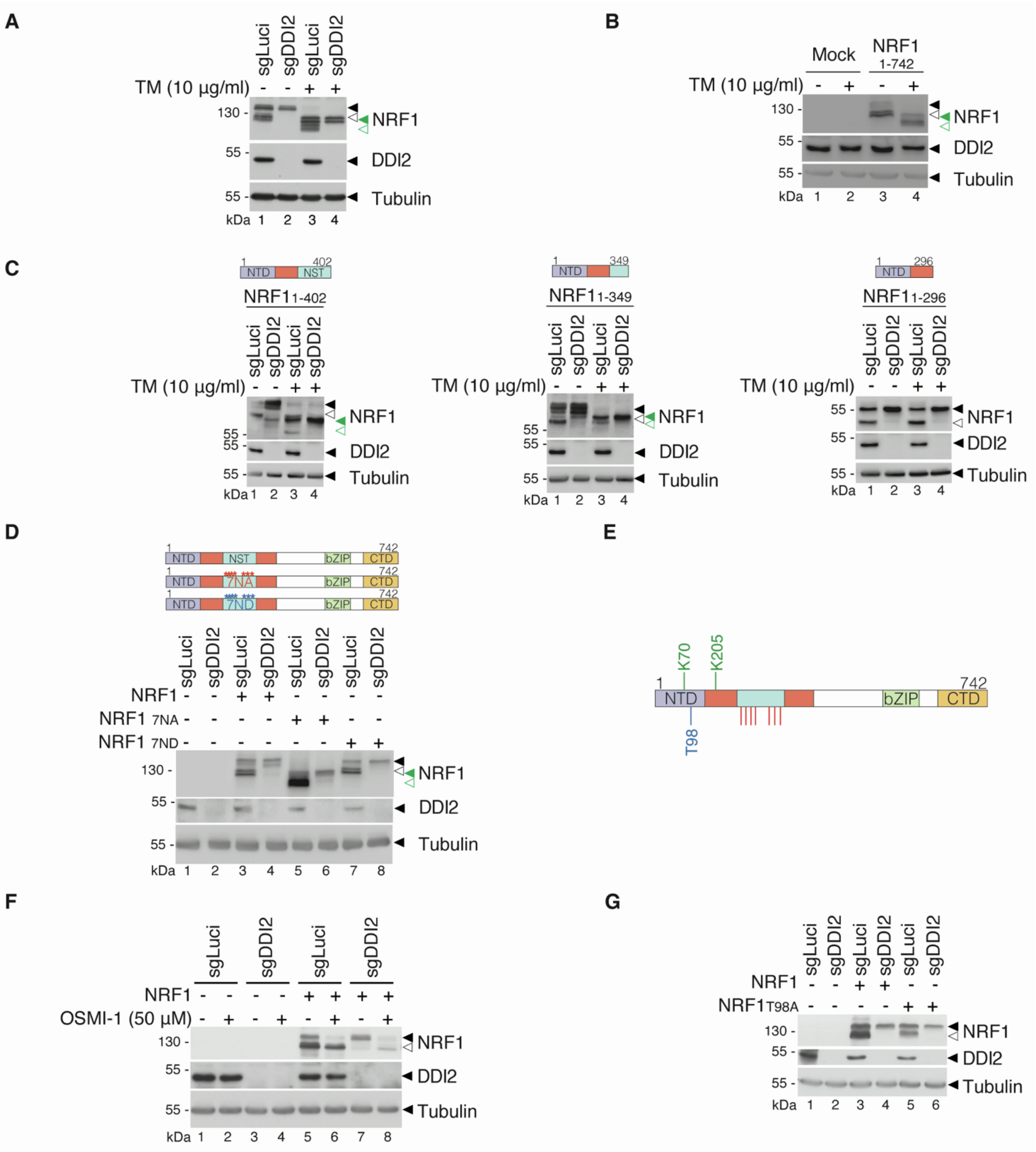

Supplemental figure 3. NRF1 is cleaved independently of its glycosylation state, related to Figure 3. (A) Endogenous NRF1 cleavage in control (sgLuci) and DDI2 knock-out (sgDDI2) ARH77 cells treated with Tunicamycin (TM) for six hours as indicated. Protein expression is measured by western blot. Tubulin is used as loading control; ◀ indicates the unprocessed NRF1 protein; ◁ indicates the cleaved NRF1 protein; ◀ indicates the deglycosylated unprocessed NRF1 protein; ◁ indicates the deglycosylated cleaved NRF1 protein. (**B**) NRF1 cleavage in HEK293T transfected with NRF1 wild-type and treated with TM for six hours. Protein expression is measured by western blot as in A. **(C)** NRF1 cleavage in control (sgLuci) and DDI2 knock-out (sgDDI2) HEK293T transfected with NRF1 deletion constructs within the C-terminus and treated with TM for six hours as illustrated on the top of the panels. Protein expression is measured by western blot as in A. **(D)** NRF1 cleavage in control (sgLuci) and DDI2 knock-out (sgDDI2) HEK293T transfected with full-length NRF1

or N-glycosylation sites mutants (7NA and 7ND) as illustrated on the top of the panel. Protein expression is measured by western blot as in A. **(E)** Schematic representation of NRF1 PTMs sites predicted by LC-MS analysis described under "Material and Methods". — indicates O-glycosylated residue; — indicate ubiquitinated residues; — indicates N-glycosylated residues. **(F)** Endogenous NRF1 cleavage in control (sgLuci) and DDI2 knock-out (sgDDI2) HEK293T cells treated with OSMI-1 for six hours as indicated. NRF1 cleavage was monitored by western blot. Tubulin is used as loading control; ◀ indicates the full-length NRF1 protein; ◁ indicates the cleaved NRF1 protein. **(G)** NRF1 cleavage in control (sgLuci) and DDI2 knock-out (sgDDI2) HEK293T transfected with NRF1 wild-type or O-glycosylation site mutant (T98A). NRF1 cleavage was monitored by western blot as in F. Western blots are representative of three (C, F), two (B, D, E) or one (G) independent experiments.

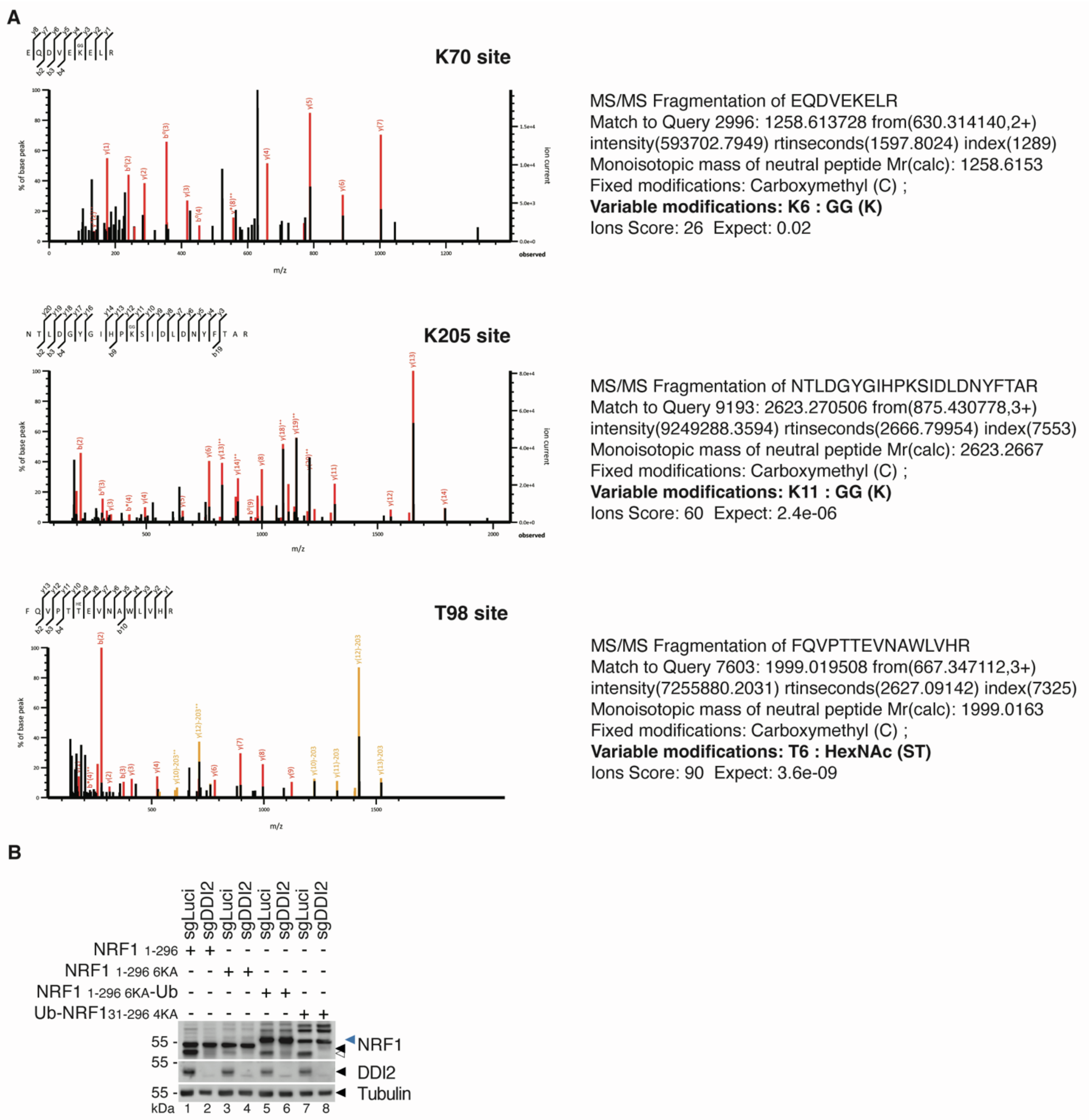

Supplemental figure 4. NRF1 is ubiquitinated at lysin 70 and 205, related to Figure 3 and S3. (A) NRF1 immunoprecipitation in control (sgLuci) and DDI2 knock-out (sgDDI2) HEK293T cells. Immunoprecipitated samples were loaded on SDS-PAGE gel. One band per condition corresponding to full-length and cleaved NRF1 respectively, were cut off and digested with trypsin for subsequent LC-MS analysis. A relevant peptide was selected for each post-translational modification and quantified based on signal intensity (precursor mass intensity). Mascot search results were imported into the MsViz software [1], which was used for validation of the PTM localization and quantitation of modified peptide intensities across samples based on extracted ion chromatograms (XIC). Peak heights in XIC traces as extracted by MSViz were used as quantitative measure. **(B)** NRF1 cleavage in control (sgLuci) and DDI2 knock-out (sgDDI2) HEK293T transfected with NRF1 (1-296) or (1-296 6KA) or fused with a ubiquitin moiety at the C-terminus, (31-296 4KA) fused with a ubiquitin moiety at the N-terminus. Protein expression is measured by western blot; ◀ indicates the unprocessed protein; ◁ indicates the cleaved protein. ◀ indicates the unprocessed NRF1 protein fused to ubiquitin moiety in N- or C-terminus. Western blots are representative of at least two independent experiments.

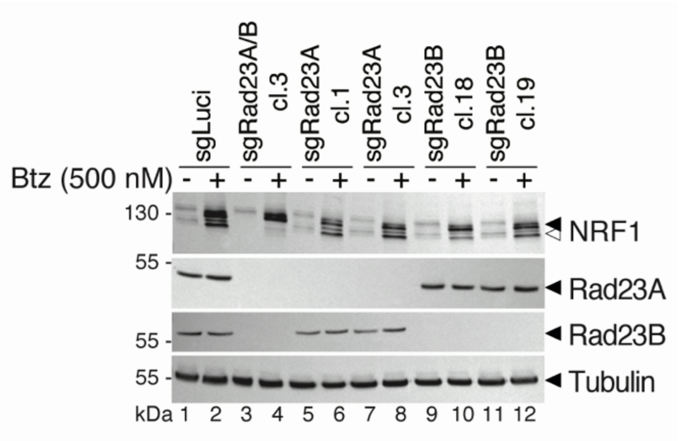

Supplemental figure 5. NRF1 cleavage requires both RAD23 paralogues, related to Figure 6. Endogenous NRF1 cleavage in control (sgLuci), double RAD23 knock-out (sgRad23A/B cl.3), RAD23A knock-out (sgRad23A cl.1 and cl.3) and RAD23B knock-out (sgRad23B cl.18 and cl.19) HEK293T cells treated with Btz for six hours as indicated. Protein expression is measured by western blot. Tubulin is used as loading control; ◀ indicates the full-length NRF1 protein; ◁ indicates the cleaved NRF1 protein.

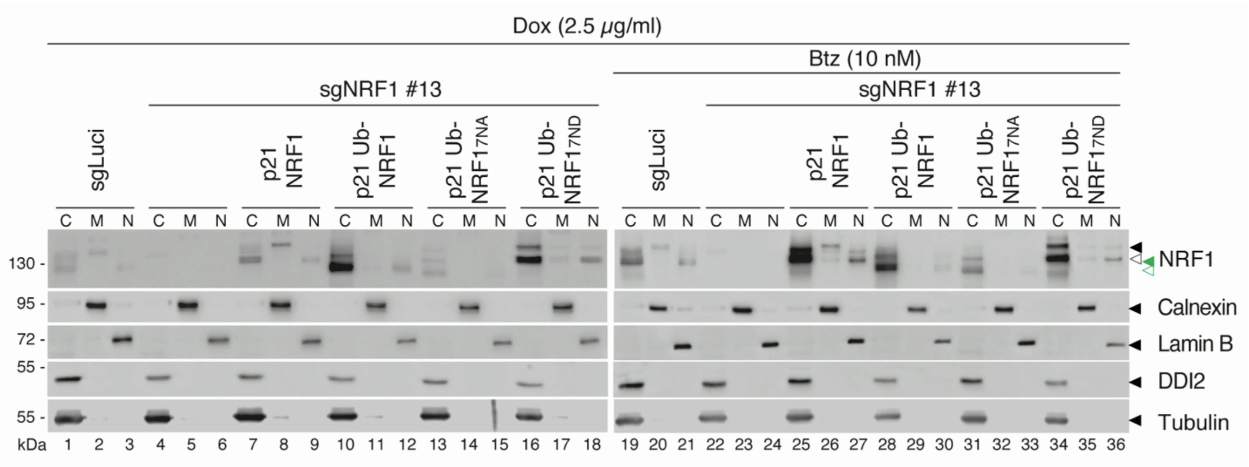

Supplemental figure 6. Ub-NRF1 constructs are cleaved in the cytosol, related to Figure 7. NRF1 cleavage in control (sgLuci), DDI2 knock-out (sgDDI2), NRF1 knock-out (sgNRF1) - or reconstituted with full-length NRF1 (p21 NRF1), or NRF1 lacking its functional NTD but fused to ubiquitin moiety in N-terminus (p21 Ub-NRF1), and with N-glycosylation sites mutated into alanine (p21 Ub-NRF1 7NA) or into aspartic acid (p21 Ub-NRF1 7ND) - upon Doxycycline, in ARH77 cells treated with Btz for six hours as indicated. Lysates were fractionated by sequential centrifugation into the membrane fraction (M: ER, Golgi, mitochondria), the nucleus (N) and the cytosol (C), respectively. Calnexin, Lamin B and Tubulin are loading and fractionation controls. Protein expression is monitored by western blot. ◀ indicates the unprocessed NRF1 protein; ◁ indicates the cleaved NRF1 protein; ◀ indicates the non-glycosylated unprocessed NRF1 protein; ◁ indicates the non-glycosylated cleaved NRF1 protein.

Table S1. List of antibodies, related to STAR Methods.

REAGENT or RESOURCE	SOURCE	IDENTIFIER
**Antibodies**		
Rabbit monoclonal anti-TCF11/NRF1	Cell Signaling Technology	Cat# 8052, RRID:AB_11178947
Rabbit monoclonal anti-SYVN1/HRD1	Cell Signaling Technology	Cat# 14773, RRID:AB_2798607
Rabbit monoclonal anti-Calnexin	Cell Signaling Technology	Cat# 2679, RRID:AB_2228381
Rabbit monoclonal anti-HA-tag	Cell Signaling Technology	Cat# 3724, RRID:AB_1549585
Human anti-alpha-Tubulin	Adipogen	Cat# AG-27B-0005, RRID:AB_2490494
Rabbit polyclonal anti-DDI2	Abcam	Cat# ab197081, RRID:AB_2928956
Rabbit monoclonal anti-Rad23A	Cell Signaling Technology	Cat# 24555, RRID:AB_2750888
Rabbit monoclonal anti-Rad23B	Cell Signaling Technology	Cat# 13525, RRID:AB_2798247
Rabbit polyclonal anti-VSV-G	Sigma-Aldrich	Cat# V4888, RRID:AB_261872
Goat polyclonal anti-Lamin B	Santa Cruz Biotechnology	Cat# sc-6217, RRID:AB_648158
Goat polyclonal anti-Mouse IgG (H+L)	Jackson ImmunoResearch Labs	Cat# 115-035-146, RRID:AB_2307392
Goat polyclonal anti-Rabbit IgG (H+L)	Jackson ImmunoResearch Labs	Cat# 111-035-144, RRID:AB_2307391
Donkey polyclonal anti-Goat IgG (H+L)	Jackson ImmunoResearch Labs	Cat# 705-035-003, RRID:AB_2340390
Donkey polyclonal anti-Human IgG (H+L)	Jackson ImmunoResearch Labs	Cat# 709-035-149, RRID:AB_2340495

Table S2. List of cell lines, related to STAR Methods.

REAGENT or RESOURCE	SOURCE	IDENTIFIER
**Cell lines**		
HEK293T sgLuci	This paper	N/A
HEK293T sgDDI2	This paper	N/A
HEK293T sgRad23A/B cl.3	This paper	N/A
HEK293T sgRad23A cl.1	L. Zaffalon	
HEK293T sgRad23A cl.3	L. Zaffalon	
HEK293T sgRad23B cl.18	L. Zaffalon	
HEK293T sgRad23B cl.19	L. Zaffalon	
HeLa sgLuci	This paper	N/A
HeLa sgDDI2 #5	This paper	N/A
ARH77 sgLuci	[2]	N/A
ARH77 sgDDI2 #30	[2]	N/A
ARH77 sgDDI2 #30 p21 Ub-NRF1 7ND	This paper	N/A
ARH77 sgNRF1 #13	[2]	N/A
ARH77 sgNRF1 #13 p21 NRF1	[2]	N/A
ARH77 sgNRF1 #13 p21 Ub-NRF1	This paper	N/A
ARH77 sgNRF1 #13 p21 Ub-NRF1 7NA	This paper	N/A
ARH77 sgNRF1 #13 p21 Ub-NRF1 7ND	This paper	N/A

Table S3. List of oligonucleotides, related to STAR Methods.

REAGENT or RESOURCE	SOURCE	IDENTIFIER
**Oligonucleotides**		
single guide RNA targeting Luciferase forward: CAC CGC TTC GAA ATG TCC GTT CGG T	[2]	N/A
single guide RNA targeting Luciferase reverse: AAA CAC CGA ACG GAC ATT TCG AAG C	[2]	N/A
single guide RNA targeting DDI2 forward: CAC CGG CTC GAA GTC GGC GTC GAC	[2]	N/A
single guide RNA targeting DDI2 reverse: AAA CGG TCG ACG CCG ACT TCG AGC C	[2]	N/A
single guide RNA targeting NRF1 forward: CAC CGC TTT CTC GCA CCC CGT TGT C	[2]	N/A
single guide RNA targeting NRF1 reverse: AAA CGA CAA CGG GGT GCG AGA AAG C	[2]	N/A
single guide RNA targeting Rad23A exon 2 forward: CAC CGT GAG TTT CTG TCC AGC CAC G	This paper	N/A
single guide RNA targeting Rad23A exon 2 reverse: AAA CCG TGG CTG GAC AGA AAC TCA	This paper	N/A
single guide RNA targeting Rad23B tr59139 exon 4 forward: CAC CGC TAG CCC AAC AGC AAC TGA C	This paper	N/A
single guide RNA targeting Rad23B tr59139 exon 4 reverse: AAA CGT CAG TTG CTG TTG GGC TAG	This paper	N/A
single guide RNA targeting HRD1 exon 4 #1 forward: TCC CGT GAA GAG TGC AAC AAA GCG G	This paper	N/A
single guide RNA targeting HRD1 exon 4 #1 reverse: AAA CCC GCT TTG TTG CAC TCT TCA C	This paper	N/A
single guide RNA targeting HRD1 exon 4 #2 forward: TCC CGG CTG AAG TCA TCC CGA AAA A	This paper	N/A
single guide RNA targeting HRD1 exon 4 #2 reverse: AAA CTT TTT CGG GAT GAC TTC AGC C	This paper	N/A
qPCR primer for GAPDH housekeeping gene forward: CGC TCT CTG CTC CTC CTG TT	[2]	N/A
qPCR primer for GAPDH housekeeping gene reverse: CCA TGG TGT CTG AGC GAT GT	[2]	N/A
qPCR primer for RPL19 housekeeping gene forward: CAA GCG GAT TCT CAT GGA ACA CAT C	This paper	N/A
qPCR primer for RPL19 housekeeping gene reverse: CTT GAT GAT CTC CTC CTT CTT GGC	This paper	N/A
qPCR primer for GABARAPL1 gene forward: CCC TCC CTT GGT TAT CAT CCA	This paper	N/A
qPCR primer for GABARAPL1 gene reverse: ACT CCC ACC CCA CAA AAT CC	This paper	N/A
qPCR primer for STYK1 gene forward: AGC GTT CTG GAC CTC AAG G	This paper	N/A
qPCR primer for STYK1 gene reverse: ATA TTG GCT CGA AAG ATG GGC	This paper	N/A
qPCR primer for CLU gene forward: CGA GAA GGC GAC GAT GAC	This paper	N/A
qPCR primer for CLU gene reverse: GGT GGA ACA GTC CAC AGA CA	This paper	N/A

1. Martin-Campos, T., Mylonas, R., Masselot, A., Waridel, P., Petricevic, T., Xenarios, I., and Quadroni, M. (2017). MsViz: A Graphical Software Tool for In-Depth Manual Validation and Quantitation of Post-translational Modifications. J Proteome Res *16*, 3092-3101. 10.1021/acs.jproteome.7b00194.

2. Op, M., Ribeiro, S.T., Chavarria, C., De Gassart, A., Zaffalon, L., and Martinon, F. (2022). The aspartyl protease DDI2 drives adaptation to proteasome inhibition in multiple myeloma. Cell Death Dis *13*, 475. 10.1038/s41419-022-04925-3.
